# Exploring Handwriting-Based Biomarkers for Alzheimer’s Disease: Identifying Discriminative Features and Tasks to Enhance Diagnostic Accuracy

**DOI:** 10.3390/diagnostics16050697

**Published:** 2026-02-26

**Authors:** Cansu Akyürek Anacur, Asuman Günay Yılmaz, Bekir Dizdaroğlu

**Affiliations:** 1 Graduate School of Natural and Applied Sciences, Karadeniz Technical University, 61080 Trabzon, Turkey; 2 Department of Computer Engineering, Bitlis Eren University, 13000 Bitlis, Turkey; 3 Department of Artificial Intelligence and Data Engineering, Karadeniz Technical University, 61080 Trabzon, Turkey; gunaya@ktu.edu.tr; 4 Department of Computer Engineering, Karadeniz Technical University, 61080 Trabzon, Turkey; bekir@ktu.edu.tr

**Keywords:** Alzheimer’s detection, ensemble learning, extended feature set, feature engineering, handwriting analysis, task reduction

## Abstract

**Background/Objectives:** This study proposes a comprehensive classification framework for the automatic detection of Alzheimer’s disease using handwriting data. An enriched feature space is constructed by combining 18 baseline features extracted from raw handwriting signals with 30 additional features derived from established handwriting analysis studies, resulting in a total of 48 features. To enhance clinical practicality, a task reduction analysis is conducted by comparing the full dataset containing 25 handwriting tasks with a reduced dataset comprising 14 selected tasks. **Methods:** The proposed framework employs a two-stage evaluation strategy involving four feature selection methods (Random Forest Feature Importance, Extreme Gradient Boosting Feature Importance, L1 Regularization and Recursive Feature Elimination), three normalization techniques (Unnormalized, Min–Max and Z-Score), and five baseline machine learning classifiers (Random Forest, Logistic Regression, Multilayer Perceptron, XGBoost and Support Vector Machines). In the second stage, a dynamic ensemble learning strategy is introduced, where the most effective classifiers are adaptively selected for each cross-validation fold and integrated using soft and hard voting schemes. **Results:** The experimental results demonstrate that reducing the number of tasks leads to an improvement in average classification accuracy from 79.47% to 81.03%, while simultaneously decreasing training time and memory consumption by approximately 40% and 35%, respectively. The highest classification performance, achieving an accuracy of 94.20%, is obtained using the Hard Ensemble combined with L1-based feature selection. **Conclusions:** These findings highlight that the joint use of enriched feature representations, task reduction, and dynamic ensemble learning provides an effective and computationally efficient solution for handwriting-based Alzheimer’s disease detection.

## 1. Introduction

Neurodegenerative diseases are characterized by their chronic and progressive nature, for which no definitive cure currently exists. The most prominent examples include Alzheimer’s disease (AD) and Parkinson’s disease (PD) [1]. These disorders are characterized by gradual deterioration of brain and nervous system functions, leading to significant impairments in cognitive ability, memory, motor control, and daily living activities. These impairments not only reduce patients’ quality of life but also place substantial social and economic burdens on families and healthcare systems [2,3].

AD is the primary cause of dementia cases worldwide. Studies have shown that approximately 60–80% of all dementia cases result from AD [4]. The disease has widespread effects, including motor coordination deficiencies, progressive decline in cognitive functions, and severe memory impairment. Patients with AD often display noticeable changes in their handwriting due to deteriorating motor coordination and reduced cognitive capacity. These handwriting alterations serve as clear indicators of how the disease affects the nervous system and motor control pathways.

Early detection of AD is critical for maintaining quality of life and facilitating timely therapeutic interventions. Current AD diagnosis protocols rely on various medical procedures and neuroimaging modalities. However, conventional diagnostic approaches are typically implemented after characteristic clinical symptoms have emerged, by which time substantial neuropathological progression has already occurred. In this context, the deterioration and changes observed in the handwriting skills of AD patients are regarded as among the early biomarkers of the disease [5].

In the literature, several studies have investigated handwriting-based classification approaches for AD detection. In one study, data collected from 130 participants performing nine distinct handwriting tasks were analyzed. By extracting the basic features commonly used in literature, the performances of widely used classification algorithms were compared [6]. Another study adopted a similar methodology and conducted a detailed analysis of 18 characteristic features extracted from 25 different handwriting tasks. The study performed both collective evaluation across all tasks and individual task-based analyses, implementing machine learning algorithms to classify individuals with AD and healthy controls [7]. In a further study, a total of 35 characteristic features were extracted from 34 different handwriting tasks, and comprehensive evaluation experiments were conducted via various classification algorithms following feature selection techniques [8].

In this study, the 18 features proposed for the diagnosis of AD in [7] were extracted from the raw data of the DARWIN (Diagnosis AlzheimeR WIth haNdwriting) dataset also introduced in [7]. To enhance the feature space, 30 additional features were incorporated, resulting in a comprehensive dataset comprising 48 features. With the expanded dataset, the effectiveness of the features in AD diagnosis was investigated. Feature selection methods were implemented to identify features with greater discriminatory power for disease diagnosis. After feature selection, multiple machine learning algorithms were applied to perform classification between AD patients and healthy controls. A comparative performance analysis was also conducted between the full feature set consisting of 48 features and the reduced feature subset obtained via feature selection techniques. The effect of feature selection on classification performance was quantitatively assessed to determine the most discriminative and informative feature subset. Moreover, potential data acquisition constraints were considered, including incomplete or suboptimal execution of the 25 handwriting tasks—particularly in later stages due to patient fatigue—as well as missing task-related files in the raw dataset. Accordingly, task-level contributions to disease classification were analyzed, and the feasibility of maintaining or improving classification accuracy via a reduced subset of handwriting tasks was systematically investigated.

The main contributions of this study are summarized as follows:

- A baseline set of 18 handwriting features is expanded by incorporating 30 additional features previously used in various handwriting recognition tasks, and their relevance for AD detection is systematically analyzed.
- Feature selection techniques are applied to identify the most informative features for distinguishing patients, with the aim of supporting the clinical interpretation of handwriting impairments associated with AD.
- On the basis of the selected features, the number of handwriting tasks is reduced from 25 to 14, with the goal of simplifying the assessment protocol and reducing the cognitive and physical burden on patients.
- Overall, the proposed approach emphasizes interpretability and clinical applicability while building upon established methodologies in the literature.

The primary contribution of this study lies in the systematic enrichment and clinical validation of the handwriting feature space for AD detection. By integrating previously unexplored descriptors with established metrics, the proposed approach captures the multidimensional characteristics of neurodegenerative motor impairment while improving model interpretability. Additionally, the task reduction strategy demonstrates that high diagnostic performance can be achieved with a more efficient and clinically feasible assessment protocol. This study emphasizes a clinically grounded, feature-centric framework that supports methodological rigor and translational applicability rather than algorithmic novelty.

## 2. Related Work

Diseases such as AD and PD fall into the category of neurodegenerative disorders for which a definitive cure has not yet been identified. Current treatment options are limited to symptom reduction and slowing disease progression. In clinical practice, these diseases are often diagnosed after characteristic symptoms appear. By this stage, the underlying pathology has usually progressed substantially. Therefore, early diagnosis and timely intervention are essential. Consequently, the need for early diagnostic strategies has led to an increase in scientific studies in this field. Alterations and deformations in handwriting are considered important indicators for the early detection of such diseases [9]. For this reason, researchers have increasingly focused on disease detection approaches based on handwriting analysis.

Stefano et al. examined handwriting analysis approaches for the early diagnosis, monitoring, and follow-up of neurodegenerative diseases and identified key topics for future research [10]. Loconsole et al. proposed a method for distinguishing patients with PD from healthy individuals by handwriting analysis [11]. Kahindo et al. extracted different types of features to group similar cognitive profiles and focused on selecting optimal features [12]. Xu and Pan proposed an ensemble learning model that combines Random Forest (RF) and Principal Component Analysis to distinguish healthy individuals from patients with PD [13]. Dentamaro et al. systematically analyzed speed-based and kinematic parameters across eight different handwriting tasks [14]. Ranjan and Swetapadma evaluated the performance of several machine learning approaches, including Artificial Neural Networks (ANN), Support Vector Machines (SVM), and k-Nearest Neighbors (k-NN), for PD detection. Their comparative analysis assessed the diagnostic effectiveness of these classification algorithms [15]. Kamble et al. analyzed static and dynamic spirals drawn by patients with PD. They compared different classification methods using kinematic variables extracted from handwriting data [16]. In the study conducted by Impedovo, feature extraction was performed using the PaHaW dataset. In this research, a new speed-based feature set was proposed to extend the baseline feature set. Prior to the classification stage, the extracted features were normalized to have zero mean and unit variance. During feature selection, the individual classification performance of each feature was evaluated, and those that achieved the highest performance were included in the refined feature set. Although various classifiers were examined in the study, the reported results were obtained via SVM [17]. Mandar et al. collected handwriting data from healthy individuals and patients using a digital pen, extracted features, and selected the first 200 features using the minimum redundancy–maximum relevance (mRMR) method. Classification models were then developed using advanced machine learning algorithms. These models were employed to distinguish between a healthy control group and patient groups [18]. Impedovo and Pirlo conducted experiments on two datasets to analyze handwriting recognition approaches. They applied classification algorithms, including SVM, Linear Discriminant Analysis (LDA), and Logistic Regression (LR), to classify PD and AD patients [19]. Cilia et al. applied the widely used Recursive Feature Elimination (RFE) method to identify the most effective features for predicting symptoms associated with cognitive impairment through handwriting analysis [20]. In another study, they proposed an ensemble model that combined the outputs of different classifiers to predict cognitive impairment [21]. D’Alessandro et al. grouped handwriting features using the sigma–lognormal model and applied seven classification algorithms. Performance was further improved through stacking and majority voting techniques [22]. Nardone et al. extracted 35 features from 34 handwriting tasks, with a particular focus on stroke-based features. After applying feature selection techniques, they conducted evaluation experiments using various classification algorithms [8]. Rohith et al. developed an MLP-based model using kinematic features from handwriting samples and showed that handwriting-based assessments could serve as promising noninvasive tools for AD screening and prognosis evaluation [23].

One of the main challenges in diagnosing AD through handwriting analysis is data scarcity. Confidentiality concerns and ethical constraints limit the creation of large-scale datasets. To address this issue, Ahmed et al. proposed a variational autoencoder (VAE)-based method for data augmentation. In this approach, synthetic data were generated from limited handwriting samples in the DARWIN dataset to create a larger and more diverse training set. These findings indicate that VAE can improve the accuracy of early AD diagnosis while protecting data privacy [24]. Singh and Chaturvedi developed a two-stage stacking model on the DARWIN dataset and used SHAP (SHapley Additive exPlanations) to improve the interpretability of the decision-making process. This method enables the identification of handwriting dynamics effective for diagnosing AD and supports the adoption of artificial intelligence (AI)–based approaches in clinical settings [25]. Salman et al. compared various machine learning and deep learning algorithms on the DARWIN dataset to investigate the effectiveness of AI-based approaches for the early detection of AD. The results showed that the stack model, in particular, offers a reliable method for the early diagnosis of AD with high accuracy and discriminative performance [26]. Bazarbekov et al. proposed an AI-based method for early AD diagnosis using handwriting movement data collected with a sensor-equipped smart pen. They evaluated various machine learning and deep learning methods and found that the combined CNN-BiLSTM model outperformed the others in distinguishing between cases. These findings suggest that handwriting-based movement data could serve as a noninvasive and clinically applicable tool for early diagnosis of AD [27]. Yao et al. proposed a multilevel information fusion approach for AD detection using handwritten data from the DARWIN dataset. The method showed stable and satisfactory performance, particularly under limited data conditions. These findings suggest that handwriting-based approaches may serve as potential tools for assessing the early stages of AD [28].

In this context, many studies have focused on improving disease recognition performance through optimal feature selection. The impact of different feature types on recognition performance has been widely examined in the literature. Motivated by these findings, this study investigates the effect of different feature sets on classification performance using the DARWIN dataset and further evaluates whether similar or higher performance can be achieved with fewer tasks.

## 3. Materials and Methods

**Figure 1:**
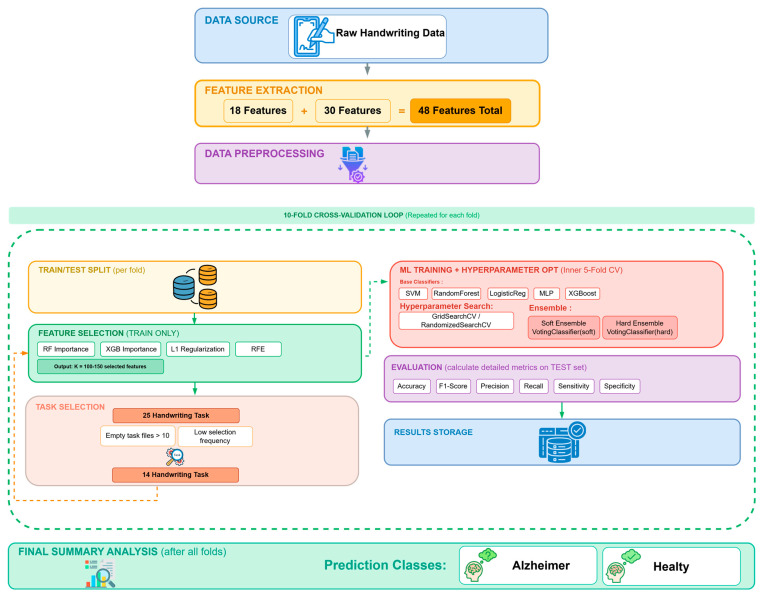
System architecture.

In this study, raw handwriting data from the DARWIN dataset [7] were analyzed for the early detection of AD. Distinctive features were extracted using mathematical techniques and transformed into numerical datasets suitable for machine learning. Feature selection methods were then applied, and the classification performance of various machine learning algorithms was comparatively evaluated. The overall system architecture is illustrated in Figure 1. Additionally, this study examines whether optimal classification performance can be achieved with fewer handwriting tasks. Classification performance was systematically evaluated on reduced task sets using task elimination strategies.

### 3.1. Dataset

In this study, the raw data from the DARWIN handwriting dataset [7] were used. The dataset includes a total of 25 different handwriting tasks collected via a graphics tablet from 89 Alzheimer’s patients and 85 healthy individuals. The tasks are structured into three main categories: memory and dictation (M), graphical drawing (G), and copying (C). Graphical tasks include horizontal/vertical lines, circles of different diameters, and complex shape-tracing tasks, which are all designed to assess participants’ basic motor control and drawing stability. Copying tasks include linguistic and visuomotor integration elements such as letters, syllables, words, reversals, postal forms, and phone numbers. Memory and dictation tasks are structured to assess short-term verbal memory, language processing skills, and sensory-motor writing production. The tasks commonly used in cognitive assessment, such as the clock drawing test, are also included in the set. This comprehensive task diversity allows for a multifaceted examination of cognitive, motor, and visuomotor performance in Alzheimer’s patients. The raw data includes the pen’s x- and y-coordinates on the graphics tablet, pen pressure, pen status (in air or on the tablet), and timestamps. But the publicly available dataset includes 18 features from each individual’s handwriting tasks, yielding 450 features per person. In this study, additional features were derived from the raw data to capture detailed handwriting dynamics and improve data representation for machine learning models.

### 3.2. Feature Extraction and Normalization

In this study, the 18 features originally extracted for AD diagnosis [7] were re-extracted from the raw data using the same methodological framework based on the x- and y-coordinates, pen pressure, pen status, and timestamps. Subsequently, commonly reported handwriting features in the literature were reviewed, and additional features frequently used in handwriting recognition and anomaly detection studies—such as those on PD, cognitive impairment, and depression—were identified and extracted from the dataset. In addition to the 18 baseline features reported in [7], 30 new features were incorporated, resulting in a total of 48 features per task and a 1200-dimensional feature vector (48 × 25) for each sample. The 48 handwriting features used for the diagnosis of AD are given in Table 1.

The first 18 features correspond to the baseline features defined in [7]. Features 19–48 were identified through a review of prior studies on handwriting-based PD detection, automatic handwriting recognition, signature verification, and neuromotor disorder assessment. All 48 features were extracted from the raw DARWIN dataset and used for the computer-aided AD diagnosis. A detailed description of the 30 additional features proposed for AD diagnosis is presented below.

Mean Azimuth (MA): MA represents the average direction angle of movement vectors during handwriting. It reflects the overall directional tendency of pen movements throughout the writing process. Positive values indicate anticlockwise movement, whereas negative values indicate clockwise movement (Equation (1)).

(1) $$MA=\left(\frac{1}{n}\right)\sum_{i=1}^{n-1}\left(\arctan\left(\frac{\Delta y_{i}}{\Delta x_{i}}\right)\cdot \frac{180}{\pi }\right)\;$$

where *n* is the total number of points, $\left(x_{i},y_{i}\right)$ represents the coordinates of the *i*-th point, $\Delta y\;=\;y_{i+1}-\;y_{i}$ and $\Delta x\;=\;x_{i+1}-\;x_{i}$.

Mean Slope (MS): MS represents the average slope of the movement vectors and indicates the overall vertical/horizontal movement tendency of the handwriting (Equation (2)). Higher values indicate steeper movements, whereas lower values indicate more horizontal movements.

(2) $$MS=\left(\frac{1}{k}\right)\sum_{i=2}^{n}\left(\frac{\left(y_{i}-y_{i-1}\right)}{\left(x_{i}-x_{i-1}\right)}\right)$$

In Equation (2), *k* represents the number of valid slopes (cases where $x_{i}-\;x_{i-1}\neq \;0$).

Total Displacement (TD): This feature represents the total distance traveled by the pen. It is calculated as the sum of all point-to-point distances (Equation (3)). TD reflects the complexity of handwriting and the level of motor activity

In Equation (3), *n* is the total number of points.

(3) $$TD=\sum_{i=2}^{n}\sqrt{\;{\left(x_{i}-x_{i-1}\right)}^{2}+{\left(y_{i}-y_{i-1}\right)}^{2}}\;\;$$

Horizontal Displacement (HD): HD represents the total absolute movement along the *X*-axis, so it measures the amount of activity in the horizontal dimension of the handwriting (Equation (4)).

(4) $$HD=\sum_{i=2}^{n}\left|\;x_{i}-x_{i-1}\right|\;\;\;$$

Vertical Displacement (VD): VD represents the total absolute movement along the *Y*-axis, so it measures the amount of activity in the vertical dimension of the handwriting (Equation (5)).

(5) $$VD=\sum_{i=2}^{n}\left|\;y_{i}-y_{i-1}\right|\;\;$$

Mean Stroke Height (MSH): MSH represents the average vertical distance between the starting point of each stroke and the point where the pen lifts into the air. It reflects the vertical consistency of the strokes (Equation (6))

(6) $$MSH=\left(\frac{1}{S}\right)\sum_{j=1}^{S}\left|\;y_{air,j}-y_{start,j}\right|\;$$

In Equation (6), *S* is the total number of strokes, *j* is the stroke index (1, 2, …, S), $y_{start,j}$ represents the Y coordinate of the first on-paper point of the *j*-th stroke, and $y_{air,j}$ represents the Y coordinate of the first in-air point at the end of the *j*-th stroke.

Mean Stroke Width (MSW): MSW represents the average horizontal distance between the starting point of each stroke and the pen-up point. It reflects the horizontal consistency of the strokes and the horizontal characteristics of the handwriting. This measure does not indicate the actual stroke width; instead, it corresponds to the difference along the *X*-axis between the stroke start point and the pen-up point (Equation (7)).

(7) $$MSW=\left(\frac{1}{S}\right)\sum_{j=1}^{S}\left|\;x_{air,j}-x_{start,j}\right|\;\;$$

In Equation (7), *S* is the total number of strokes, *j* is the stroke index (1, 2, …, S), $x_{start,j}$ is the X coordinate of the first on-paper point of the *j*-th stroke, and $x_{air,j}$ is the X coordinate of the first in-air point at the end of the *j*-th stroke.

Mean Centroid Distance (MCD): MCD is defined as the average distance between the centers of gravity of consecutive strokes, indicating the spatial consistency of letter or word placement (Equation (8)).

(8) $$MCD=\left(\frac{1}{S-1}\right)\sum_{j=1}^{S-1}\sqrt{\;{\left({\bar{x}}_{j+1}-{\bar{x}}_{j}\right)}^{2}+{\left({\bar{y}}_{j+1}-{\bar{y}}_{j}\right)}^{2}}$$

In Equation (8), ${\bar{x}}_{j}=\;\left(\frac{1}{n_{j}}\right)\sum_{i}\in S_{j},$ where *x_j_* is the X coordinate of the *j*-th stroke center, ${\bar{y}}_{j}=\;\left(\frac{1}{n_{j}}\right)\sum_{i}\in S_{j}$, where *y*_j_ is the Y coordinate of the *j*-th stroke center, *n_j_* is the number of points in the *j*-th stroke, and *S* is the total number of strokes.

Mean Minimum Distance between Strokes (MMDS): This feature represents the average distance between the closest points of two consecutive strokes. It reflects the physical proximity between strokes and the degree of crowding in handwriting. For each stroke pair, distances between all points of the first stroke and all points of the second stroke are calculated, and the minimum distance is selected (Equation (9)).

(9) $$MMDS=\left(\frac{1}{S-1}\right)\sum_{j=1}^{S-1}\min\left\{\;\sqrt{\;{\left(x_{2}-x_{1}\right)}^{2}+{\left(y_{2}-y_{1}\right)}^{2}}:\;\left(x_{1},y_{1}\right)\in \;S_{j},\;\left(x_{2},y_{2}\right)\in \;S_{j+1}\right\}\;\;$$

Mean Stroke Endpoint Distance (MSED): MSED represents the average distance between the endpoint of one stroke and the starting point of the next stroke. It indicates the consistency of transitions between letters (Equation (10)).

(10) $$MSED=\left(\frac{1}{S-1}\right)\sum_{j=1}^{S-1}\sqrt{\;{\left(x_{end,j}-x_{start,j+1}\right)}^{2}+{\left(y_{end,j}-y_{start,j+1}\right)}^{2}}\;\;\;\;\;$$

In Equation (10), $x_{end,j}$ and $y_{end,j}$ represent the endpoints of the *j*-th stroke, $x_{start,j+1}$ and $y_{start,j+1}$ represent the starting points of the (*j* + 1)-th stroke, and *S* is the total number of strokes.

Mean Vertical Alignment (MVA): MVA expresses the average difference between the *Y*-axis coordinates of the end points of consecutive strokes. It is a measure of the consistency of the handwriting’s vertical arrangement (Equation (11)).

(11) $$MVA=\left(\frac{1}{S-1}\right)\sum_{j=2}^{S}\left|\;y_{end,j}-y_{end,j-1}\right|\;\;\;$$

In Equation (11), *S* is the total number of strokes, $y_{end,j-1}$ is the Y-coordinate of the end point of the (*j* − 1)-th stroke, and $y_{end,j}$ is the Y-coordinate of the end point of the *j*-th stroke.

Mean Horizontal Alignment (MHA): This feature represents the average difference between the *X*-axis coordinates of consecutive stroke endpoints. It reflects the consistency of horizontal flow and linear alignment in handwriting (Equation (12)).

(12) $$MHA=\left(\frac{1}{S-1}\right)\sum_{j=2}^{S}\left|\;x_{end,j}-x_{end,j-1}\right|\;$$

In Equation (12), *S* is the total number of strokes, $x_{end,j-1}$ is the X-coordinate of the end point of the (*j* − 1)-th stroke, and $x_{end,j}$ is the X-coordinate of the end point of the *j*-th stroke.

Horizontal Shannon Entropy (HSE): HSE is computed from the distribution of *X*-axis coordinates and quantifies the randomness and variability of horizontal pen movements. It serves as a measure of horizontal flow consistency and linear alignment in handwriting. Low entropy values indicate that the pen moves more regularly along the *X*-axis (Equation (13))

(13) $$HSE\left(X\right)=-\sum_{i=1}^{n}\left[p\left(x_{i}\right){\times \log}_{2}p\left(x_{i}\right)\right]\;\;$$

In Equation (13), $p\left(x_{i}\right)=\frac{count\left(x_{i}\right)}{n}$ is the probability distribution of the value $x_{i}$, where *n* is the total number of points.

Vertical Shannon Entropy (VSE): VSE measures the randomness and variability of vertical pen movements using the distribution of *Y*-axis coordinates. The entropy value is calculated from the occurrence frequencies of distinct Y-coordinate values. Low entropy values indicate that the pen moves more regularly along the *Y*-axis (Equation (14)).

(14) $$VSE\left(Y\right)=-\sum_{i=1}^{n}\left[p\left(y_{i}\right)\times \log_{2}p\left(y_{i}\right)\right]\;$$

In Equation (14), $p\left(y_{i}\right)=\frac{count\left(y_{i}\right)}{n}$ is the probability distribution of the value *y_i_*, and *n* is the total number of points.

Horizontal Rényi Entropy (2) (HRE_2_): This feature is a generalized form of Shannon entropy and is computed using the parameter α = 2. It contains information about the second moment of the X-coordinate distribution. Because it assigns lower weights to rare values compared to Shannon entropy, it is also referred to as collision entropy in the literature. This type of entropy is used to identify repeating patterns within a signal (Equation (15)).

(15) $$HRE_{2\left(X\right)}=-\log_{\left(2\right)}\sum_{i=1}^{n}p{\left(x_{i}\right)}^{2}$$

In Equation (15), α = 2 is the Rényi entropy parameter, $p\left(x_{i}\right)$ is the probability (less weight to rare values), and (1 − α) = −1 is the normalization factor.

Horizontal Rényi Entropy (3) (HRE_3_): The Rényi entropy calculated with the parameter α = 3 contains higher-order moment information compared to both Shannon entropy and Rényi entropy with α = 2. It also assigns lower weights to rarely observed values. This type of entropy is used to identify dominant patterns within a signal (Equation (16)).

(16) $$HRE_{3\left(X\right)}=-\left(\frac{1}{2}\right)\log_{\left(2\right)}\sum_{i=1}^{n}p{\left(x_{i}\right)}^{3}\;\;\;\;$$

In Equation (16), α = 3 is the Rényi entropy parameter, $p\left(x_{i}\right)\;$ is the probability, and (1 − α) = −2 is the normalization factor.

Vertical Rényi Entropy (2) (VRE_2_): The Rényi entropy calculated with the parameter α = 2 for the Y-coordinate sequence provides an analysis based on the second moment of vertical movement patterns. This measure enables the identification of repetitions and regular structures in vertical movements. Low entropy values indicate a more consistent vertical movement pattern concentrated around specific Y levels (e.g., row handwriting consistency), whereas high entropy values reflect a more irregular and dispersed distribution of vertical movements along the *Y*-axis (Equation (17)).

(17) $$VRE_{2\left(Y\right)}=-\log_{\left(2\right)}\sum_{i=1}^{n}p{\left(y_{i}\right)}^{2}$$

In Equation (17), α = 2 is the Rényi entropy parameter, $p\left(y_{i}\right)\;$ is the probability distribution of the Y coordinate values and (1 − α) = −1 is the normalization factor.

Vertical Rényi Entropy (3) (VRE_3_): The Rényi entropy computed with α = 3 for the Y-coordinate sequence captures higher-order statistical characteristics beyond those represented by Shannon entropy and Rényi entropy with α = 2. This formulation places greater emphasis on dominant vertical movement values while reducing the influence of rarely observed Y-coordinate occurrences. As a result, it provides a clearer representation of the underlying structure and primary characteristics of the vertical movement distribution (Equation (18)).

(18) $$VRE_{3\left(Y\right)}=-\left(\frac{1}{2}\right)\log_{\left(2\right)}\sum_{i=1}^{n}p{\left(y_{i}\right)}^{3}$$

In Equation (18), α = 3 is the Rényi entropy parameter, $p\left(y_{i}\right)$ represents the probabilities (focusing on dominant patterns), and (1 − α) = −2 is the normalization factor.

*X*-axis Total Energy (CE_X_): CE_X_ quantifies the total energy by computing the sum of the squared X-coordinate values, thereby representing the overall activity level and kinetic energy of handwriting along the horizontal axis. This parameter serves as an indicator of the horizontal spatial dispersion of the handwriting and reflects the extent of the pen’s horizontal movement capacity (Equation (19)).

(19) $$CE_{X}=\sum_{i=1}^{n}x_{i}^{2}$$

In Equation (19), *x_i_* is the X-coordinate of the *i*-th point, and *n* is the total number of points.

*Y*-axis Total Energy (CE_Y_): CE_Y_ represents the total energy obtained by summing the squared Y-coordinate values, providing a quantitative measure of the overall activity level and kinetic energy of handwriting along the vertical axis. This parameter characterizes the vertical spatial dispersion of the handwriting trajectory and reflects the extent of the pen’s vertical movement dynamics (Equation (20)).

(20) $$CE_{Y}=\sum_{i=1}^{n}y_{i}^{2}$$

In Equation (20), *y_i_* is the Y coordinate of the *i*-th point, and *n* is the total number of points.

*X*-axis Teager–Kaiser Energy (TKE_X_): The Teager–Kaiser operator-based energy measure is used to estimate instantaneous energy changes in the X-coordinate signal. It is applied to analyze motor control disorders and tremor-like irregularities in the horizontal dimension. This operator enables the detection of sudden variations in horizontal movement patterns, including frequency modulations and amplitude changes (Equation (21)).

(21) $$TKE_{X}=\sum_{i=2}^{n-1}\left(\;x_{i}^{2}-x_{i-1}x_{i+1}\right)\;$$

In Equation (21), *x_i_* is the X-coordinate of the *i*-th point, and *n* is the total number of points.

*Y*-axis Teager–Kaiser Energy (TKE_Y)_: TKE_Y_ is computed to estimate instantaneous energy changes in the Y-coordinate signal. It is used to analyze motor control irregularities and tremor-like movements in the vertical dimension. This operator enables the detection of sudden changes, frequency modulations, and amplitude variations in vertical movement patterns (Equation (22)).

(22) $$TKE_{Y}=\sum_{i=2}^{n-1}\left(\;y_{i}^{2}-y_{i-1}y_{i+1}\right)\;\;$$

In Equation (22), *y_i_* is the Y coordinate of the *i*-th point, and *n* is the total number of points.

*X*-axis CE Signal-to-Noise Ratio (SNRCE_X_): The signal-to-noise ratio (SNR) is calculated based on the energy values derived from the squared X-coordinates. It quantitatively evaluates the consistency of energy levels along the horizontal axis. The ratio is obtained by dividing the average energy level by the energy variability. High SNR values indicate more consistent and regular horizontal movement energy profiles, suggesting a more stable motor control structure. In contrast, low SNR values reflect increased variability in energy distribution and more irregular horizontal movement patterns (Equation (23)).

(23) $$SNRCE_{X}=\frac{\mu (x_{i}^{2})}{\sigma (x_{i}^{2})}\;\;\;\;\;\;\;$$

In Equation (23), μ is the mean and σ  is the standard deviation.

*Y*-axis CE Signal-to-Noise Ratio (SNRCE_Y)_: The SNR is calculated using the energy values derived from the squared Y-coordinates. It quantitatively evaluates the consistency of energy levels along the vertical axis. The ratio is obtained by dividing the average energy level by the energy variability, providing a measure of vertical motor control consistency. This parameter is used to investigate the effects of neurological disorders on vertical movement control (Equation (24)).

(24) $$SNRCE_{Y}=\frac{\mu (y_{i}^{2})}{\sigma (y_{i}^{2})\;}\;\;\;$$

In Equation (24), μ is the mean and σ  is the standard deviation.

*X*-axis TKE Signal-to-Noise Ratio (SNRTKE_X_): The SNR calculated based on the Teager–Kaiser energy values on the *X*-axis is defined as the ratio of the mean of the TKE values to their standard deviation. This feature quantitatively assesses the consistency of instantaneous energy changes observed in horizontal movement patterns, enabling the measurement of motor control irregularities (Equation (25)).

(25) $$SNRTKE_{X}=\frac{\mu \left(x_{i}^{2}-x_{i-1}x_{i+1}\right)}{\sigma \left(x_{i}^{2}-x_{i-1}x_{i+1}\right)\;\;}$$

In Equation (25), *μ* and *σ* are the mean and standard deviation of the Teager–Kaiser energy values, respectively.

*Y*-axis TKE Signal-to-Noise Ratio (SNRTKE_Y_): The SNR calculated from Teager–Kaiser energy values along the *Y*-axis quantitatively evaluates the consistency of instantaneous energy variations in vertical movement patterns. It is used to analyze motor control disorders in the vertical dimension (Equation (26)).

(26) $$SNRTKE_{Y}=\frac{\mu \left(y_{i}^{2}-y_{i-1}y_{i+1}\right)}{\sigma \left(y_{i}^{2}-y_{i-1}y_{i+1}\right)\;\;}$$

In Equation (26), μ and σ are the mean and standard deviation, respectively, of the TKE values on the *Y*-axis.

Speed Standard Deviation (SSD): This feature represents the standard deviation of instantaneous velocity values calculated throughout the handwriting process. It is used to identify characteristic velocity irregularities associated with neurological disorders. Instantaneous velocity is computed by dividing the Euclidean distance between two consecutive points by the corresponding time difference, considering only valid time intervals (*dt* > 0). This parameter quantitatively measures the variability in speed values. Low values indicate more consistent speed control and stable motor performance, while high values represent increased speed variability and motor control irregularities (Equation (27)).

(27) $$SSD=\sqrt{\;\left(\frac{1}{m-1}\right)\sum_{i=1}^{m}{\left(\;speed_{i}-\mu_{speed}\right)}^{2}}\;\;$$

In Equation (27), $speed_{i}=\;\frac{\sqrt{\;{\left(x_{i+1}-\;x_{i}\right)}^{2}+\;{\left(y_{i+1}-\;y_{i}\right)}^{2}}}{dt_{i}}$ represents the speed during the *i*-th time interval, and $\mu_{speed}$ represents the average speed.

Pressure Standard Deviation (PSD): PSD represents the standard deviation of all pressure values measured throughout the handwriting process. It quantitatively reflects the variability in the pressure applied to the pen. This calculation includes all pressure values measured when the pen is on the paper (*o_i_* = 1) and when it is in the air (*o_i_* = 0). Lower values indicate more consistent pressure control and more stable hand–arm coordination, while higher values reflect increased motor control impairments (Equation (28)).

(28) $$PSD=\sqrt{\;\left(\frac{1}{n-1}\right)\sum_{i=1}^{n}{\left(\;z_{i}-\mu_{z}\right)}^{2}}\;\;\;\;\;\;$$

In Equation (28), *z_i_* is the pressure value at the *i*-th point, $\mu_{z}$ is the average pressure, and *n* is the total number of points (including both *o_i_* = 0 and *o_i_* = 1).

Altitude: This feature represents the mean elevation angle of vectors formed between consecutive points. It is defined as the average angle between each vector and the *Z*-axis (pressure). Positive values indicate increasing pressure trends, while negative values reflect pressure reduction. This parameter characterizes pen dynamics and pressure control behavior in the vertical pressure dimension. (Equation (29)).

(29) $$Altitude=\left(\frac{1}{k}\right)\sum_{i=1}^{k}\left(\arcsin\left(\frac{dz_{i}}{\sqrt{\;{\left(dx_{i}\right)}^{2}+{\left(dy_{i}\right)}^{2}+{\left(dz_{i}\right)}^{2}}}\right)\cdot \frac{180}{\pi }\right)$$

In Equation (29), $dz_{i}=\;z_{i+1}-\;z_{i}$ is the change in the pressure axis, and $dx_{i}=\;x_{i+1}-\;x_{i}$ and $dy_{i}=\;y_{i+1}-\;y_{i}$ are the changes in horizontal and vertical coordinates.

Horizontal Intrinsic Shannon Entropy (H_IMF1_): This feature represents the Shannon entropy of the first intrinsic mode function (IMF1) obtained through Empirical Mode Decomposition (EMD) of the X-coordinate signal. The EMD method decomposes the signal into components with different frequency characteristics, and the highest-frequency component (IMF1) is selected for analysis. The entropy of this component provides a quantitative measure of high-frequency noise and signal irregularities. This parameter is used to detect motor control tremors and fine motor movement impairments (Equation (30)).

(30) $$H_{IMF1\left(X\right)}=-\sum_{i=1}^{n}p\left(IMF1,i\right)\log_{\left(2\right)}p\left(IMF1,i\right)$$

Here, IMF1 is the first intrinsic mode function of the EMD of the signal X, and *p* (IMF1, *i*) is the probability distribution of the IMF1 values.

The literature review identified three distinct feature sets employed in previous studies [7,8,33] utilizing the DARWIN dataset. The proposed 48-feature dataset was evaluated against feature sets reported in previous studies to identify similarities, differences, and overlapping parameters. The results of this comparison are summarized in Table 2. The table shows that all 18 features defined in study [7], considered the foundational work for the DARWIN dataset, are included in the present study. Among the proposed 48 features, 26 features have not previously been used in the literature related to the DARWIN dataset and therefore introduce novel characteristics. The remaining 22 features overlap with earlier studies, including 18 shared with study [7], 7 with study [41], and 8 with study [8]. The proposed feature set was designed by integrating methodological insights and strengths identified in prior handwriting-based studies for AD diagnosis.

The feature set was designed to incorporate geometric and temporal analyses in addition to fundamental kinematic measurements. The 26 newly introduced features, constituting more than half of the proposed feature set, aim to facilitate a more comprehensive characterization of handwriting patterns associated with irregularities, energy distribution, and alignment behaviors observed in AD. This multi-component analytical framework enables the quantitative characterization of subtle and fine-grained handwriting alterations, extending and enhancing the insights provided by existing approaches.

After constructing the 18- and 48-feature sets, experimental evaluations were conducted under three preprocessing strategies: unscaled data, Min–Max normalization, and Z-score standardization. These approaches were selected due to their widespread use in machine learning and medical signal processing, as well as their representation of distinct normalization paradigms. The unscaled configuration served as a baseline by preserving the original feature distributions. Min–Max normalization applied range-based scaling to the [0, 1] interval, whereas Z-score standardization performed distribution-based scaling by centering features at zero mean with unit variance. All methods are computationally efficient and require no additional hyperparameter tuning, thereby supporting reproducibility and methodological consistency.

### 3.3. Feature Selection

At this stage, the reduced feature subsets were generated using feature selection methods. The resulting feature subsets were evaluated using multiple classification algorithms, and evaluation metrics were analyzed to assess the effects of scaling and feature selection strategies on classification performance. During the feature selection stage, sub-feature sets were derived using Random Forest (RF) Feature Importance (RF Importance), Extreme Gradient Boosting (XGBoost) Feature Importance, L1 regularization, and Recursive Feature Elimination (RFE) methods.

Feature selection was employed instead of dimensionality reduction techniques such as Principal Component Analysis (PCA) to ensure clinical interpretability within the handwriting-based AD detection framework. The extracted features, including writing speed, pen pressure, and other kinematic and temporal parameters, correspond to quantifiable motor functions that may reflect cognitive decline and neuromotor impairment associated with AD. Preserving these original variables enables direct assessment of the relationship between specific handwriting dynamics and disease status.

In contrast, PCA projects the original feature space onto orthogonal components that are linear combinations of multiple variables. While this transformation can mitigate multi-collinearity and reduce dimensionality, the resulting components lack clear physiological or behavioral meaning, limiting their clinical interpretability. Given that one of the primary objectives of this study was not only predictive performance but also the identification of clinically meaningful digital biomarkers, feature selection was considered a more appropriate methodological choice.

The feature selection methods employed in this study are explained in detail in the following subsections.

#### 3.3.1. RF_Importance

RF is an ensemble learning method based on the collective predictions of multiple decision trees [42]. In this study, feature selection was performed using the decrease in Gini impurity contributed by each feature during decision tree splits. The Gini index measures the heterogeneity of class distribution at a node and is defined as follows:

(31) $$Gini\left(t\right)=\;1\;-\;\sum_{c=1}^{C}p_{c}^{2}$$

Here, *C* represents the number of classes, and *p_c_* represents the proportion of samples belonging to class *c* at the *t*-node. A feature’s global importance score is calculated based on the average Gini impurity reduction provided by that feature across all splits made in all trees. In this method, features are ranked according to their importance scores, and the *k* features with the highest scores are selected.

#### 3.3.2. XGBoost Feature Importance

The XGBoost algorithm [43] uses trees trained sequentially with the gradient boosting framework. Each new tree focuses on correcting the mistakes of the previous trees. In this study, feature importance was evaluated using the weight metric, defined as the frequency with which each feature was selected as a splitting criterion across all trees:

(32) $$Weight\left(x_{j}\right)=\;\sum_{T=1}^{N_{T}}Count\left(T,\;x_{j}\right)$$

In the equation, *N_T_* represents the total number of trees, and *Count*(*T*, *x_j_*) represents the number of times feature *x_j_* is used as the split criterion in the *T*-th tree. Due to the algorithmic structure of gradient boosting, features that contribute more significantly to the model tend to be selected more frequently. Features were ranked based on normalized weight scores, and the top *k* features were selected.

#### 3.3.3. L1 Regularization

L1 regularization (Lasso—Least Absolute Shrinkage and Selection Operator) is a regularization method that adds the sum of the absolute values of the coefficients as a penalty term to the logistic regression model [44]. A key characteristic of this method is its ability to achieve automatic feature selection by forcing some feature coefficients to become exactly zero during optimization. The objective function to be minimized for logistic regression with L1 regularization is expressed as follows:

(33) $$\min_{w}\left(L\left(w\right)+\;\lambda \;\sum_{j=1}^{p}\left|w_{j}\right|\right)$$

Here, $L\left(w\right)$ represents the cross-entropy loss function, w is the vector of feature coefficients, λ is the hyperparameter controlling the regularization strength, and *p* is the total number of features. In multi-class classification problems, a separate coefficient vector is obtained for each class by applying the One-vs-Rest (OvR) strategy. In this case, the global importance score of a feature was calculated by taking the average of the absolute values of its coefficients across all classes:

(34) $$Score\left(x_{j}\right)=\;\left(\frac{1}{C}\right)\sum_{c=1}^{C}\left|\;w_{c\;j}\right|\;\;\;\;$$

Here, C represents the number of classes, and *w_cj_* represents the coefficient of the *j*-th feature for the *c*-th class. Features are ranked in descending order based on these scores, and the *k* features with the highest scores are selected.

#### 3.3.4. RFE

RFE is a wrapper-based feature selection method [45]. The algorithm iteratively eliminates the least important features to determine the optimal feature subset. At each iteration, a model (Random Forest in this study) is trained on the current feature set, feature importance scores are calculated, and a specified proportion of the lowest-ranked features (determined by the step parameter) is removed. This process is repeated until the desired number of *k*-clusters is reached:

(35) $$S*=\;argmin_{S\;\subseteq \;F,\;\left|S\right|=\;k}E\left(f_{S}\right)$$

Here, *F* represents the entire feature set, S* is the optimal *k*-feature subset, $E\left(\cdot \right)$ is the model error, and *f_S_* is the model trained with the subset *S*. The main advantage of RFE is that it updates feature importances by retraining the model at each step, thus considering dependencies between features.

### 3.4. Classification

In this study, five different machine learning algorithms (Support Vector Machines (SVM), RF, Logistic Regression (LR), Multi-Layer Perceptron (MLP), and XGBoost) were employed for the classification task using the selected feature subsets. The classification methods used in this study are explained in the following subsections.

#### 3.4.1. SVM

The main objective of SVM is to identify an optimal separating hyperplane that maximizes the margin between classes, defined by the distance between the hyperplane and the closest data points (support vectors) [46]. However, real-world datasets generally exhibit non-linearly separable class distributions. For this reason the soft-margin SVM approach, which tolerates class overlap, was utilized in this study. The optimization problem solved for the soft-margin SVM is defined below:

(36) $$\underset{w,b,\xi }{\min}\left(\frac{1}{2}{\left|\left|w\right|\right|}^{2}+\;C\;\sum_{i=1}^{n}\xi_{i}\right)\;$$

$$s.t.\;\;y_{i}\left(\;w^{T}\varphi \left(x_{i}\right)+b\;\right)\geq \;1-\xi_{i},\;\;\;\xi_{i}\geq \;0\;,\;i=1,\ldots ,n$$

In this formulation, *w* ∈ ℝ*^d^* represents the normal vector of the hyperplane, *b* ∈ ℝ is the bias (offset) term, *x_i_* ∈ ℝ*^d^* is the feature vector of the *i*-th training sample, *y_i_* ∈ {−1, +1} is the class label, ξ*_i_* ≥ 0 is the slack variable for the *i*-th example, and *n* is the total number of training samples. In the original space, data that is not linearly separable becomes separable in a high-dimensional feature space using the kernel function φ(·). In this study, two different kernel functions were used: (1) the linear kernel and (2) the Radial Basis Function (RBF) kernel.

#### 3.4.2. RF

RF is an ensemble learning algorithm that combines the predictions of multiple decision trees using bootstrap aggregation (bagging) and random feature selection approaches [42]. In the model, each decision tree is trained independently using bootstrap-sampled subsets of the training data. The final decision is determined by majority voting on the outputs of all trees:

(37) $$\hat{y}\;=\;mode\left\{\;h_{1\left(x\right)},\;h_{2\left(x\right)},\;\ldots \;,\;h_{T\left(x\right)}\right\}$$

where *h_t(x)_* represents the prediction of the *t*-th tree, *T* is the total number of trees, and *mode*{·} is the most frequent value (majority voting).

#### 3.4.3. LR

LR is a probabilistic classification method that uses a linear decision boundary [47]. In this approach, the probability of an instance belonging to a specific class is modeled using the sigmoid (logistic) function. The logistic function used for the binary classification problem is defined as follows:

(38) $$P(y=1\;|\;x)\;=\;1/(\;1\;+\;exp(\;-\;(\;w^{T}x\;+\;b\;)\;)\;)\;\;\;\;\;\;$$

Here, $P\left(y=1\;|\;x\right)$ represents the probability of belonging to class 1, *w* is the learnt weight vector, *x* is the feature vector, and *b* is the bias term. Model parameters are learnt by minimizing the negative loglikelihood (cross-entropy) function.

#### 3.4.4. MLP

MLP is a feedforward artificial neural network architecture [48]. It consists of an input layer, one or more hidden layers, and an output layer. The mathematical expression used for a single hidden layer is presented below:

(39) $$h\;=\;f\left(\;W^{\left\{\left(1\right)\right\}x}+\;b^{\left\{\left(1\right)\right\}}\right)\;\;$$

(40) $$\hat{y}=g\left(\;W^{\left\{\left(2\right)\right\}h}+b^{\left\{\left(2\right)\right\}}\right)$$

where *h* represents the hidden layer output, *f*(·) is the hidden layer activation function (ReLU, tanh, or sigmoid), *g*(·) is the output activation function (softmax), *W*^(1)^ and *W*^(2)^ are the weight matrices, *b*^(1)^ and *b*^(2)^ are the bias vectors.

#### 3.4.5. XGBoost

XGBoost is an optimized ensemble learning algorithm that is based on the gradient boosting framework [43]. In this approach, weak learners (mostly decision trees) are sequentially added to the model to create a strong learner. Each new tree focuses on modelling the residual errors caused by the previous model. In this respect, XGBoost models have an additive structure as follows:

(41) $${\hat{y}}_{i\left(t\right)}=\;{\hat{y}}_{i\left(t-1\right)}+\;\eta \;\cdot \;f_{t\left(x_{i}\right)}\;\;\;\;\;$$

where *ŷ_i_*^(*t*)^ represents the prediction at the *t*-th iteration, *f_t_* is the newly added tree, *η* is the learning rate, and *x_i_* is the feature vector of the *i*-th example. XGBoost prevents overfitting by adding L1 and L2 regularization terms to the loss function:

(42) $$L\left(t\right)=\;\sum_{i=1}^{n}l\left(\;y_{i},\;{\hat{y}}_{i\left(t\right)}\right)+\;\sum_{k=1}^{t}\Omega \left(\;f_{k}\right)$$

where *l*(·) represents the loss function (cross-entropy/logloss), *Ω*(*f_k_*) is the tree complexity penalty, *n* is the number of samples, and *t* is the current iteration.

### 3.5. Task Selection

The DARWIN handwriting dataset was collected using a graphics tablet and includes a total of 25 different handwriting tasks from 89 individuals diagnosed with AD and 85 healthy controls. Analysis of the dataset indicated that a considerable number of participants had empty or missing data files for some tasks. A total of 153 empty task files were detected, including 54 (35.3%) from healthy controls and 99 (64.7%) from individuals with AD. These findings indicate that individuals diagnosed with AD encountered greater difficulty in completing the tasks.

During the task selection stage, 924 distinct model configurations were evaluated for AD diagnosis based on a feature matrix comprising 174 samples and 1200 features (25 tasks × 48 features). The configurations included combinations of three normalization strategies, seven classifiers (five standalone algorithms and two ensemble methods), four feature selection techniques, and eleven feature subset sizes (3 × 7 × 4 × 11). The analysis examined which handwriting tasks contributed to the selected features in the highest-performing configurations. Based on these findings, tasks with a high number of missing data files (more than 10) and low feature selection frequency were excluded from the dataset.

### 3.6. Ensemble Learning

Ensemble learning integrates the predictions of multiple base classifiers to enhance model robustness and generalization. In this study, two voting-based ensemble strategies were employed. Base classifiers were selected based on their performance in the inner cross-validation stage, with only those exceeding a predefined performance threshold incorporated into the ensemble framework. The final class label was determined by aggregating the predictions of the selected base classifiers through voting, thereby enhancing generalization performance.

#### 3.6.1. Soft Voting

Soft voting is decided by averaging the probability estimates of each base classifier. The final probability for each class is calculated as follows:

(43) $$P\left(y=c|x\right)=\;\left(\frac{1}{M}\right)\sum_{m=1}^{M}P_{m\left(y=c|x\right)}\;$$

Here, *M* represents the number of models in the ensemble, and $P_{m\left(y\;=\;c\;|\;x\right)}$ indicates the probability predicted by the *m*-th model for class *c* given *x*.

#### 3.6.2. Hard Voting

Hard voting (majority voting) takes the mode (most frequent value) of the class predictions from each base model:

(44) $$\hat{y}\;=\;mode\left(\;h_{1\left(x\right)},\;h_{2\left(x\right)},\;\ldots \;,\;h_{M\left(x\right)}\right)\;\;\;$$

Here, *h_m_*(*x*) represents the class prediction of the *m*-th model. Hard voting is suitable for classifiers that do not produce probability estimates or provide reliable probabilities.

### 3.7. Use of Generative Artificial Intelligence Tools

During the preparation of this manuscript, generative artificial intelligence tools (ChatGPT (GPT-5.0), OpenAI, San Francisco, CA, USA; and Claude (Sonnet-4.5), Anthropic, San Francisco, CA, USA) were used to assist with language refinement, debugging suggestions during code development, comparative table structuring, and improving the technical articulation of performance result discussions.

The tools were not used to generate original data, conduct statistical analyses, develop algorithms, or independently interpret experimental findings. All experimental procedures, analytical processes, scientific evaluations, and final interpretations were conducted and validated exclusively by the authors.

The authors carefully reviewed and revised all AI-assisted outputs and assume full responsibility for the integrity, accuracy, and originality of this manuscript.

## 4. Experimental Results and Discussion

This section presents the experimental results obtained through a multi-stage evaluation process. Initially, features associated with AD diagnosis were extracted from the raw dataset. Subsequently, relevant features and tasks were identified using multiple feature selection algorithms. Finally, different classification algorithms were applied to the optimized feature set, and their performances were comparatively evaluated using multiple evaluation metrics.

### 4.1. Experimental Settings

The proposed framework was developed using Python 3.13.5. All simulations and model training processes were executed on a Windows 11 Pro (64-bit) platform, running on an Intel^®^ Core™ i9-13900HX processor (2.20 GHz) with 64 GB DDR5 RAM (5200 MT/s) and a 12 GB dedicated GPU. The hardware configuration ensured stable execution of large-scale feature engineering, repeated k-fold cross-validation, and ensemble learning procedures without memory bottlenecks. GPU resources were employed for accelerated numerical computations when supported by the utilized libraries.

In this study, feature extraction from the raw dataset was performed in two stages. In the first stage, 18 features were extracted following the methodology described in [7]. In the second stage, 30 additional features commonly used in the literature for handwriting analysis were computed. As a result, a total of 48 features were obtained for each task. Since the same set of features was extracted for all tasks, the final feature set had dimensions of 174 × 1200 (25 tasks × 48 features).

During feature set preparation, three dataset configurations were generated: an un-normalized dataset containing raw feature values, a min–max-normalized dataset scaled to the range [0, 1], and a *Z*-score-normalized dataset standardized to zero mean and unit variance. This design enables a comparative evaluation of the effects of different normalization strategies on classification performance.

Subsequently, four different feature selection algorithms were applied to the feature sets. Feature selection was conducted independently for each normalization strategy. To determine the optimal number of features, eleven different values of *k* (100, 105, 110, 115, 120, 125, 130, 135, 140, 145, and 150) were evaluated. The impact of varying the number of selected features on classification performance was systematically analyzed. To reduce the risk of overfitting and enhance the model’s generalization capability, 10-fold stratified cross-validation was employed during the feature selection process. This approach ensures a balanced partitioning of the dataset into training and testing subsets while preserving the proportional distribution of healthy controls and AD patients in each fold. For each experimental configuration, the selected feature subsets were recorded, and the consistency and frequency of feature selection for different normalization strategies were analyzed.

The performance of all classification algorithms was evaluated using a nested cross-validation strategy. In the outer loop, the dataset was partitioned into training and testing sets using 10-fold stratified cross-validation, ensuring that the class distribution in each fold preserved the proportions of the original dataset. In the inner loop, hyper parameter optimization was conducted on the training set using 5-fold cross-validation with either GridSearchCV or RandomizedSearchCV. This nested evaluation framework enables an objective assessment of the models’ generalization performance while reducing the risk of overfitting during hyper parameter selection.

### 4.2. Task Reduction Strategy

Several task files in the dataset contained empty or missing data. Out of a total of 153 empty task files, 35.3% corresponded to healthy control subjects, while 64.7% were associated with individuals diagnosed with AD. A detailed task-wise distribution of empty files is presented in Table 3. As shown, Task 19 had the highest number of empty files (28), followed by Task 21 (13), Task 25 (12), and Tasks 20, 22, and 24 (10 each). A notable level of data insufficiency was observed in tasks ranging from 19 to 25.

Feature selection was applied to the 174 × 1200 dataset using 308 model configurations (7 classifiers × 4 feature selection methods × 11 feature subset sizes) for each normalization strategy. Tasks contributing to the top-performing configurations were then examined, and their selection frequencies across normalization strategies are summarized in Table 4.

Table 4 indicates that some tasks were consistently selected more frequently than others regardless of normalization strategy. Task 9 showed the highest selection frequency across all configurations, emphasizing its strong contribution to top-performing models. Tasks 7 and 8 also displayed stable and relatively high selection frequencies, whereas tasks such as 19 and 22 were rarely selected, suggesting lower relevance to the final feature subsets. Overall, task selection patterns were largely invariant to normalization strategy.

A low negative correlation (−0.231) was observed between the number of empty task files and the frequency of task selection, suggesting that data deficiency may be associated with reduced feature discriminability. Despite having a relatively high number of empty files (9), Task 23 exhibited comparatively high selection frequencies across all normalization strategies. This observation indicates that Task 23 retains discriminative relevance in the feature selection process even in the presence of missing data, distinguishing it from other tasks with similar levels of data deficiency.

As a result of these analyses, tasks exhibiting a high rate of empty files (more than 10 empty files) combined with low selection frequencies (e.g., Tasks 15, 18, 19, and 22) were excluded from the dataset. As a result, 11 out of the original 25 tasks were eliminated, leaving a reduced set of 14 tasks, namely Tasks 1, 3, 4, 5, 7, 9, 10, 11, 13, 14, 16, 17, 23, and 25. In the initial phase of the study, a feature set of size 174 × 1200 was constructed by extracting 48 features from each of the 25 tasks. Following task reduction, 48 features were extracted from the remaining 14 tasks, resulting in a task-reduced feature set of dimensions 174 × 672. The feature selection and classification results obtained using the 25-task and 14-task datasets are presented and compared in the subsequent subsections.

### 4.3. Evaluation of Discriminative Features for AD Diagnosis

#### 4.3.1. Feature Selection Analyses for the 25-Task Feature Set

A detailed analysis of the features selected in the highest-performing configurations under three normalization strategies for the 25-task feature set is presented in Table 5. The feature selection outcomes obtained from the unnormalized, Z-score-, and Min–Max-normalized data show a high level of consistency across methods, indicating that the proposed feature extraction framework is robust to variations in data scaling. Moreover, the results suggest that the most influential features effectively capture aspects of motor control, movement stability, and information processing complexity reflected in the handwriting behavior of individuals with AD.

Notably, features introduced in this study account for approximately 65% of the top 20 most frequently selected features, highlighting their potential discriminative value in AD diagnosis. These features are SNRCE_X_, MA, MS, MVA, Altitude, TKE_X_, MSW, HSE, SNRTKE_Y_, PSD, MMDS, MHA and MSH. The features SNRCE_X_, MA, MS, MVA, and Altitude represent handwriting dynamics and movement quality metrics such as movement direction accuracy, stroke slope, movement consistency and signal-to-noise ratio.

The consistently high selection frequency of SNRCE_X_ across all normalization techniques suggests a pronounced degradation in the signal-to-noise characteristics of hand movements associated with AD. This finding may be associated with impairments in motor planning, increased micro-level movement irregularities, and reduced sensorimotor coordination observed in the early stages of AD. The high ranking of metrics related to in-air micro-movements, such as MVA and MJA, suggests that AD influences not only the writing execution phase but also motor planning and coordination processes. This finding supports the hypothesis that the disease influences movement preparation processes in addition to motor execution.

On the other hand, the low ranking of basic physical measures such as TT, PM, and TD suggests that simple parameters related to speed, duration, or pressure are insufficient for effective AD diagnosis alone. The results demonstrate that features capturing non-linear movement behavior (e.g., TKE_X_, SNRTKE_X_, SNRTKE_Y_, SNRCE_X_, and MJA), entropy-based complexity measures (e.g., HSE, VSE, and HRE_3_), and dynamic signal characteristics (e.g., AT, PT, Altitude, GMRT, GMRTP, and GMRTA) play a substantially more discriminative role. These findings indicate that handwriting-based AD detection systems should prioritize advanced signal processing techniques, energy-based movement analyses, and statistical complexity metrics over basic kinematic measures.

Among the feature selection combinations yielding the highest performance across all normalization strategies, the selection frequency of the newly added features was analyzed. On average, 113.33 features were selected across the best-performing configurations (110 for the unnormalized setting, 120 for Min–Max normalization, and 110 for Z-score normalization). Of these, an average of 66.87 features corresponded to those introduced in this study (67.05 for unnormalized, 66.5 for Min–Max, and 67.05 for Z-score normalization). These findings demonstrate that features capturing fine-grained motor behavior and signal characteristics (such as micro-kinematic properties, directional consistency, stroke coordination, signal-to-noise ratio, and entropy-related measures) were more frequently selected than traditional handwriting metrics based on time, speed, and pressure in high-performing models.

#### 4.3.2. Feature Selection Analyses for the 14-Task Feature Set

An overview of the features identified in the top-performing configurations across three normalization strategies for the 14-task feature set is shown in Table 6. Notably, 65% of the 20 most frequently selected features comprise novel features introduced in this study, suggesting that these new features demonstrate discriminative capacity for AD classification, even within the feature set developed for the reduced 14-task.

The findings reveal that the SNRCE_X_ feature exhibits the strongest discriminative power with a selection frequency of 6.75 across all normalization strategies. The consistently high selection frequencies of item orientation and movement characteristics—including MA, MS and MJA—across normalization techniques indicate that these parameters constitute significant biomarkers for neurodegenerative diseases. Z-score normalization demonstrates divergent behavior compared to other techniques, particularly for scale-dependent properties such as kinetic energy (TKE_X_) and pressure variability (PV).

Furthermore, features derived from the *X*-axis systematically exhibit higher selection frequencies than their *Y*-axis counterparts, suggesting that horizontal writing dynamics more effectively capture disease-related motor impairments. Among the pressure-related features, PSD exhibited a substantially higher selection frequency than PM, indicating that motor control inconsistencies are more prominently captured through variability-related structures rather than absolute magnitude measures. Analysis of feature selection frequencies for the reduced-task dataset shows that newly introduced features account for 62.54% of all selected features, highlighting their substantial contribution to the discriminative feature space.

#### 4.3.3. Clinical Interpretation of Discriminative Handwriting Features

**Figure 2:**
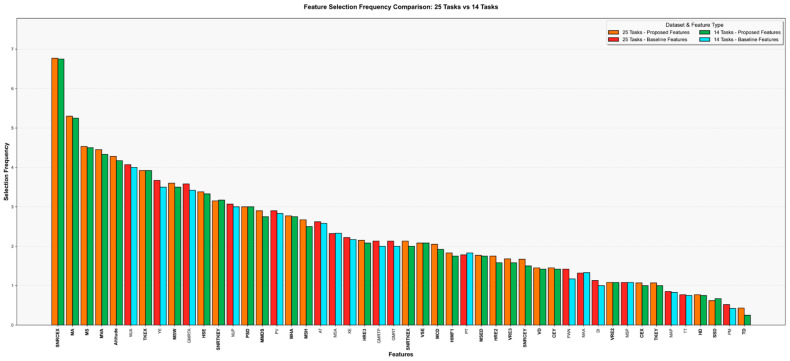
Selection frequencies of 48-features for 25-Task and 14-Task datasets.

The selection frequency results in Figure 2 shed light on the clinical relevance of the top-ranked features. Seven of the ten most frequently selected features (SNRCE_X_, MA, MS, MVA, Altitude, TKE_X_, MSW) were proposed in this study, while the remaining three (MJA, YE, GMRTA) belong to the baseline feature set originally defined in [7]. The newly introduced features, highlighted in bold in the graph, are predominantly positioned in the upper and middle ranks. This distribution suggests that the proposed features not only complement existing features but also provide direct discriminative contributions. In particular, signal-to-noise ratio measures, kinematic movement descriptors, and newly defined entropy-based criteria emerge as key components supporting model performance.

Based on the motor and cognitive functions they capture, the features can be categorized into three distinct groups. The first group is associated with motor execution and tremor, comprising SNRCE_X_, TKE_X_, MJA, and GMRTA. SNRCE_X_ quantifies the proportion of structured movement relative to noise within the horizontal trajectory. Its top ranking aligns with the systematic review by Koppelmans et al. [49], which reported cortical thinning and disruptions in cerebello–thalamo–cortical connectivity in AD and MCI populations. TKE_X_ relies on the Teager–Kaiser operator, which is effective at picking up rapid frequency and amplitude changes linked to the extrapyramidal motor symptoms observed in AD [50]. MJA quantifies the abruptness of directional changes during in-air hand movements, which primarily depend on internal motor planning rather than visual guidance. Recent studies have identified this variable as one of the strongest predictors of cognitive decline [51]. GMRTA characterizes oscillatory tremor during in-air movements, providing a friction-independent measure associated with basal ganglia and cerebellar function [18].

The second group of features relates to spatial organization and movement planning: MA, MS, MVA, YE, and MSW. Directional consistency (MA) and slope regularity (MS) both require coordinated visuospatial planning and proprioceptive feedback. A systematic review encompassing 91 studies reported that visuospatial characteristics constitute one of the most prominently impaired domains of handwriting in AD [52]. MVA and MSW capture vertical alignment between strokes and horizontal span within individual strokes, respectively. They both decline with increasing spatial disorganization in handwriting as the disease progresses [53,54]. YE represents the vertical extent of the written output and is associated with amplitude scaling regulated by basal ganglia and cortical loops. These spatial features are particularly informative, as parietal and medial temporal regions involved in spatial processing are among the earliest cortical areas affected in AD [55].

Altitude stands as the only pressure-related feature in the top ten. Unlike conventional pressure magnitude metrics, it represents the angular relationship between planar pen displacement and the pressure axis, reflecting the coordination between applied force and movement direction. This distinction matters given the contradictory findings in the literature on whether writing pressure increases or decreases in AD [56]. The dominance of these novel features over simpler kinematic descriptors suggests that advanced signal-level characterization can reveal subtle motor and cognitive disturbances not captured by basic measures.

On the other hand, the low ranking of basic physical measures such as TT, PM, and TD suggests that simple parameters related to speed, duration, or pressure are insufficient for effective AD diagnosis. The results demonstrate that features capturing non-linear movement behavior (e.g., TKE_X_, SNRTKE_X_, SNRTKE_Y_, SNRCEX, and MJA), entropy-based complexity (e.g., HSE, VSE, and HRE_3_), and dynamic signal characteristics (e.g., AT, PT, Altitude, GMRT, GMRTP, and GMRTA) offer significantly higher discriminative value compared to conventional descriptors.

### 4.4. Performance Evaluation of Feature Sets with Classification Algorithms

For both the 25-task and 14-task feature sets, a systematic feature selection and classification process was applied to datasets normalized using three distinct normalization strategies. The feature selection stage employed four different algorithms, with the hyperparameter *k* (number of selected features) evaluated across 11 distinct values (100, 105, 110, 115, 120, 125, 130, 135, 140, 145, and 150) for each algorithm. All resulting feature subsets were subsequently assessed using seven machine learning classifiers. This experimental design yielded 308 unique configurations per normalization strategy (4 feature selection algorithms × 11 *k* values × 7 classifiers). In the classification stage, in addition to five individual machine learning methods, classification performance was further enhanced through ensemble approaches employing both hard and soft voting schemes.

#### 4.4.1. Classification Performance Using the 25-Task Feature Set

The classification framework was structured as a sequential two-stage pipeline. In the first stage, five base classifiers (SVM, RF, LR, MLP, and XGBoost) were trained independently for each fold, generating individual performance metrics through direct prediction on the test set. In the second stage, an ensemble learning mechanism was implemented. Specifically, the inner cross-validation (inner-CV) performance of the base models was evaluated, and classifiers satisfying a predetermined performance threshold were dynamically selected on a per-fold basis. The ensemble stage employed voting-based aggregation of the selected classifiers’ outputs: in the soft ensemble approach, class probabilities generated by individual models were combined with equal weighting to construct the final probability distribution, from which the class with maximum probability was predicted. When soft ensemble performance exceeded a specified threshold, a hard ensemble strategy was applied to the same classifier subset, wherein predicted class labels were aggregated through majority voting to determine the final classification. This approach yields more robust and generalizable classification performance by establishing a dynamic ensemble architecture that adapts to data distribution characteristics while preserving the fold-specific performance of individual base classifiers. Table 7 presents the top 15 model configurations ranked by accuracy on the unnormalized dataset.

For the unnormalized dataset, the highest classification accuracy of 94.20% and F1-score of 94.19% were achieved using 110 features selected through L1 regularization combined with a hard ensemble classifier. The best model demonstrated well-balanced performance with a sensitivity–specificity difference of only 0.34 percentage points. The most stable model configuration, exhibiting the lowest standard deviation (3.11), utilized 120 features with L1 regularization and hard ensemble classification, yielding an accuracy of 91.96%. Notably, all top 15 model configurations employ hard ensemble classification, demonstrating that ensemble learning methods can achieve robust performance without normalization preprocessing. L1 regularization emerged as the predominant feature selection technique, appearing in 10 of the 15 top-performing configurations.

Table 8 presents the average individual performance of each classifier across all model configurations evaluated on the unnormalized dataset. Analysis reveals that the hard ensemble method achieves the highest performance, with a mean accuracy of 87.13% and a mean F1-score of 87.07%. The ensemble models (hard and soft) demonstrate a mean accuracy of 83.06%, representing a 4.97 percentage point improvement over the mean accuracy of individual classifiers (78.10%). These findings indicate that ensemble approaches yield more robust and accurate predictions by leveraging the complementary strengths of constituent models.

Table 9 presents a comparative performance analysis of feature selection methods across all model combinations evaluated on the unnormalized dataset. L1 regularization consistently demonstrates higher performance, achieving the highest mean accuracy (80.90%) and mean F1-score (80.62%), with a maximum accuracy of 94.20% indicating substantial diagnostic potential under optimal conditions. RF Importance exhibits comparably high performance while demonstrating enhanced stability with a relatively low standard deviation. Conversely, RFE-RF and XGB Importance methods yield limited performance with lower mean accuracy values. The performance differential across feature selection methods is 2.54 percentage points, representing a moderate variation.

For the min–max-normalized dataset, the highest classification accuracy of 93.07% and F1-score of 93.05% were achieved using 120 features selected through L1 regularization combined with hard ensemble classification. Notably, this top-performing model also demonstrates maximum stability, exhibiting the lowest standard deviation (2.71) among all configurations. Table 10 presents the top 15 model configurations ranked by classification performance. As can be seen from the table, all top 15 model configurations employ hard ensemble classification. L1 regularization was employed in 60% (9/15) of the top-performing configurations, establishing it as the predominant feature selection technique.

Table 11 presents the average individual performance of each classifier across all model configurations evaluated on the Min–Max-normalized dataset. Analysis reveals that the hard ensemble method achieves the highest performance, with mean accuracy of 87.25% and mean F1-score of 87.19%. The ensemble models (hard and soft) demonstrate an average accuracy rate of 83.03%, representing a 5.02 percentage point improvement compared to the average performance of individual classifiers at 78.01%. These findings demonstrate that ensemble approaches yield more robust and accurate predictions by combining the strengths of individual models.

Table 12 presents a comparative performance analysis of feature selection methods across all model combinations evaluated on the Min–Max-normalized dataset. Analysis of feature selection methods reveals that L1 Regularization consistently outperforms alternative approaches across multiple performance metrics. L1 regularization achieves the highest mean accuracy (80.90%) and mean F1-score (80.61%), while also demonstrating maximum accuracy (93.07%), indicating substantial diagnostic potential under optimal conditions Although L1 regularization exhibits slightly higher variability (standard deviation: 4.22) compared to RF Importance (3.60), its performance advantage persists. RFE Random Forest and XGBoost importance methods yield comparatively modest results, with mean accuracies of 78.61% and 78.27%, respectively. Notably, L1 regularization achieves the highest mean sensitivity (84.47%), suggesting strong capability in correctly identifying positive cases, which is particularly important for clinical diagnostic applications.

For the *Z*-score-normalized dataset, the highest classification accuracy of 92.81% and F1-score of 92.80% were achieved using 110 features selected through L1 regularization combined with hard ensemble classification. Notably, this optimal model also demonstrates maximum stability, exhibiting the lowest standard deviation (3.05) among all configurations. Table 13 presents the top 15 model configurations ranked by classification performance. Analysis reveals that all top 15 model configurations employ hard ensemble classification. L1 regularization emerged as the predominant feature selection technique, appearing in 9 of the 15 top-performing configurations.

Table 14 presents the average performance of each classifier across all model configurations evaluated on the Z-score-normalized dataset. It can be seen from the table that, the hard ensemble method achieves the highest performance, with mean accuracy of 87.14% and mean F1-score of 87.08%. The average accuracy of ensemble models 82.97% is 4.9 percentage points higher than the average performance of single classifiers, which is 78.04%. This demonstrates that ensemble approaches produce more robust and accurate predictions by combining the strengths of different models.

Table 15 presents a comparative performance analysis of feature selection methods across all model combinations evaluated on the Min–Max-normalized dataset. Analysis of Table 15 reveals that L1 regularization consistently outperforms other approaches across multiple performance metrics. This method achieves the highest mean accuracy (80.90%), mean F1-score (80.63%), and mean sensitivity (84.35%). Moreover, the maximum accuracy of 92.81% indicates that L1-based feature selection can yield highly successful results under optimal parameter configurations.

Table 16 presents a comparative analysis of the optimal model configurations achieved under each normalization strategy. The unnormalized dataset yields the highest classification accuracy (94.20%) and F1-score (94.19%) using 110 L1-regularized features with hard ensemble classification, demonstrating well-balanced sensitivity–specificity performance with only 0.35 percentage point difference. The min–max-normalized dataset achieves 93.07% accuracy with 120 features and exhibits the highest sensitivity (97.78%), though at the cost of reduced specificity (88.89%), resulting in an 8.89 percentage point imbalance that may indicate overfitting to the positive class. This configuration also demonstrates the lowest standard deviation (2.71), suggesting maximum stability. The Z-score-normalized dataset attains 92.81% accuracy with 110 features, presenting the most balanced sensitivity–specificity trade-off (2.43 percentage point difference) among normalization methods while maintaining comparable stability (standard deviation: 3.05). Notably, mean accuracy across all model configurations remains remarkably consistent across normalization strategies (79.45–79.52%), indicating that normalization primarily affects peak performance rather than average model behavior. These findings suggest that while the unnormalized approach yields marginally higher maximum accuracy, the choice of normalization strategy critically influences the sensitivity–specificity balance, with important implications for clinical deployment where false negative and false positive rates carry different consequences.

#### 4.4.2. Classification Performance Using the 14-Task Feature Set

The feature set, generated by reducing the number of tasks to 14 and normalized using three distinct normalization techniques, was evaluated using the same classification methodology applied to the feature sets comprising all tasks.

For the unnormalized dataset, the highest classification performance was achieved using the Hard Ensemble classifier in combination with the RFE Random Forest feature selection algorithm, yielding an accuracy of 91.42% with 150 selected features. Table 17 presents the top 15 model configurations that attained the highest accuracy values on the unnormalized dataset.

Table 18 presents the average individual performance of each classifier across all model configurations evaluated on the unnormalized dataset. Among all experiments, the Hard Ensemble classifier achieved the highest average performance, with an average accuracy of 87.55% and an average F1-score of 87.48%. In addition, this method also attained the highest maximum accuracy value (91.42%) among all evaluated classifiers. The XGBoost and Soft Ensemble classifiers exhibited comparatively stable performance across the experiments. RF and SVM classifiers yielded moderately balanced results, whereas the performance of the MLP and LR models remained relatively limited on this dataset.

A comparative performance analysis of feature selection methods across all model combinations evaluated on the unnormalized dataset is presented in Table 19.

The comparative performance results of the four feature selection methods evaluated in this study reveal notable differences among the approaches. L1 Regularization achieved the highest Mean Accuracy and Mean F1-score values and exhibited the lowest standard deviation in accuracy, indicating the most stable and reliable performance. The RF Importance method attained the highest Mean Sensitivity, demonstrating superior effectiveness in correctly identifying the positive class. In contrast, although the RFE Random Forest method achieved high maximum accuracy values in certain folds, it displayed considerable performance variability, reflected by a wide performance range and high variance. The XGB Importance method showed the weakest overall performance, with lower Mean Accuracy and Mean F1-score values compared to the other methods.

On the Min–Max-normalized dataset, the combination of the Hard Ensemble classifier and the L1 regularization feature selection method achieved an accuracy of 92.16% using 120 selected features. The top 15 model configurations yielding the best classification performance are presented in Table 20. The results in the table show that all of the top 15 model configurations were constructed using the Hard Ensemble classifier, indicating a consistently high performance across all evaluated combinations. Among the feature selection methods, L1 Regularization was the most frequently employed approach, being selected in 7 out of the 15 models. Models incorporating L1 regularization generally achieved higher accuracy values and more balanced performance across the evaluation metrics. Furthermore, the highest F1-score reported in the table (92.03) was obtained by the model using 120 features selected via L1 Regularization. Overall, these results demonstrate that the Hard Ensemble classifier attains its best performance when combined with L1 Regularization, representing the most successful model configuration within the scope of this study.

The average performances of each classifier for all model configurations tested on the Min–Max-normalized dataset are presented in Table 21. When examining the table, it is clear that the Hard Ensemble method demonstrates the highest performance among the classifiers. The Hard Ensemble significantly outperformed all other models in terms of Mean Accuracy (87.63%) and Mean F1-Score (87.57%) also produced the highest Max Accuracy value in the table at 92.16%. This finding reveals that the Hard Ensemble algorithm is the most stable model in terms of both overall classification performance and inter-class balance. Following the Hard Ensemble, XGBoost (81.96%) and Soft Ensemble (81.44%) showed moderate success but performed significantly worse than the Hard Ensemble, especially in terms of Mean Sensitivity and Mean Specificity values. The RF algorithm performed at a similar level to these two models in terms of accuracy (81.52%) and offered a more stable structure with lower variance. SVM, LR, and MLP classifier, on the other hand, yielded more limited results in terms of performance.

Table 22 shows a comparative performance of feature selection methods, considering all model combinations.

The results show that L1 Regularization performed the most successfully and was the most balanced among the feature selection methods, with the highest Mean Accuracy (82.49%) and Mean F1-Score (82.25%). While RF Importance is strong in correctly capturing the positive class with high Mean Sensitivity, it is weaker in terms of Mean Specificity. While RFE RandomForest produces high accuracy in some cases, it is the most unstable method due to having the highest Std Accuracy value. XGB Importance, on the other hand, yielded the lowest Mean Accuracy and Mean F1-Score values among the four methods. Overall, L1 Regularization is the most stable and effective feature selection approach for this problem.

The highest performance on the Z-score-normalized dataset was achieved by using the Hard Ensemble classifier together with the RF Importance feature selection algorithm, resulting in an accuracy rate of 91.26% with 125 features. Table 23 shows the top 15 model configurations with the best classification performance. An examination of the table reveals that the Hard Ensemble classifier is employed in all top 15 highest-performing model configurations. With respect to feature selection methods, L1 Regularization is the most frequently used approach, appearing in 6 out of 15 models, followed by RF Importance (5/15) and RFE Random Forest (3/15). Among the models, the highest F1-score (91.23%) was achieved by the combination of the Hard Ensemble classifier and RF Importance with 125 selected features.

Table 24 presents the average performance of each classifier across all model configurations evaluated on the Z-score-normalized dataset. The results indicate that the Hard Ensemble classifier outperformed the other evaluated classifiers. This method achieved the highest Mean Accuracy (87.41%), Mean F1-score (87.33%), and Maximum Accuracy (91.26%), demonstrating strong classification performance and balanced class representation. The XGBoost, RF, and Soft Ensemble classifiers exhibited moderate performance levels, whereas the SVM, LR, and particularly the MLP classifier yielded lower accuracy values and weaker class discrimination. Overall, the Hard Ensemble classifier emerged as the most successful and stable approach for this dataset.

Table 25 shows a comparative performance of feature selection methods, considering all model combinations. The results indicate that, among the evaluated feature selection methods, L1 Regularization achieved the most successful and balanced performance, yielding the highest Mean Accuracy (82.35%) and Mean F1-score (82.10%). Although the RF Importance method demonstrated better performance in identifying the positive class, as reflected by its higher Mean Sensitivity (85.78%), it exhibited reduced class balance due to lower Mean Specificity compared to L1 Regularization. In contrast, the RFE Random Forest and XGB Importance methods showed weaker and more variable performance, characterized by lower accuracy values and higher variance. Overall, L1 Regularization emerged as the most stable and effective feature selection method for this dataset.

Table 26 presents a comparative analysis of the optimal model configurations achieved under each normalization strategy. When comparing the classification performance metrics obtained for the three different normalization strategies, it was observed that the performance achieved with unnormalized data generally yielded balanced results. In this case, it is observed that the model achieved high Accuracy (91.42%) and F1-score (91.38%) values. Additionally, the sensitivity value was measured at 88.54% and the specificity value at 94.44%; the high specificity value indicates that the model is successful in correctly identifying healthy individuals (true negatives). The Sensitivity–Specificity Balance (5.90) value indicates that the model provides an acceptable balance between the two classes. The low standard deviation value (2.90) supports the model’s ability to produce stable results.

The Min–Max normalization method achieved the highest accuracy (92.16%) and the most balanced sensitivity–specificity ratio. The sensitivity value was measured as 91.67% and the specificity value as 92.59%. The fact that these two metrics are very close to each other (Sens-Spec Balance: 0.93) indicates that the model performs well in terms of class balance. This situation reveals that the model demonstrates a balanced performance in both correctly identifying Alzheimer’s patients (true positive) and correctly identifying healthy individuals (true negative). Nevertheless, the lowest standard deviation value (2.90) indicates that this is the most stable normalization in terms of consistency.

The Z-Score normalization method, on the other hand, achieved the highest Sensitivity value (96.88%). This indicates that the model has a higher tendency to correctly identify Alzheimer’s patients (true positive). However, the specificity rate (86.11%) decreased, and the Sens-Spec difference (10.76) increased, indicating a rise in the false positive rate. So, while this method is advantageous in capturing the patient class, it is relatively weak in distinguishing healthy individuals. While the model’s accuracy (91.26%) and F1-score (91.23) are high, the imbalance between sensitivity and specificity is noteworthy. The standard deviation value (2.90) indicates that it produces stable results at the same level as other methods.

### 4.5. Comparative Analysis of Classification Performance Between the 25-Task and 14-Task Datasets

This section presents a comparative analysis of results obtained from feature selection applied to a 25-task dataset and an optimized 14-task dataset. A total of 1848 model configurations were evaluated under three normalization strategies (unnormalized, Min–Max, and Z-score), with 924 models assessed for each dataset. Comparative results for the 25-task and 14-task datasets are presented in Table 27.

The results indicate that the 14-task dataset achieved higher mean accuracy values than the 25-task dataset across all normalization strategies (e.g., unnormalized: 81.03% vs. 79.52%). Lower standard deviation values also suggest more stable classification performance for the 14-task dataset. However, the highest peak accuracy was obtained with the 25-task dataset, reaching 94.20% using the Hard Ensemble classifier with L1 regularization under the unnormalized setting. In comparison, the highest accuracy for the 14-task dataset was 92.16% with Min–Max normalization.

**Figure 3:**
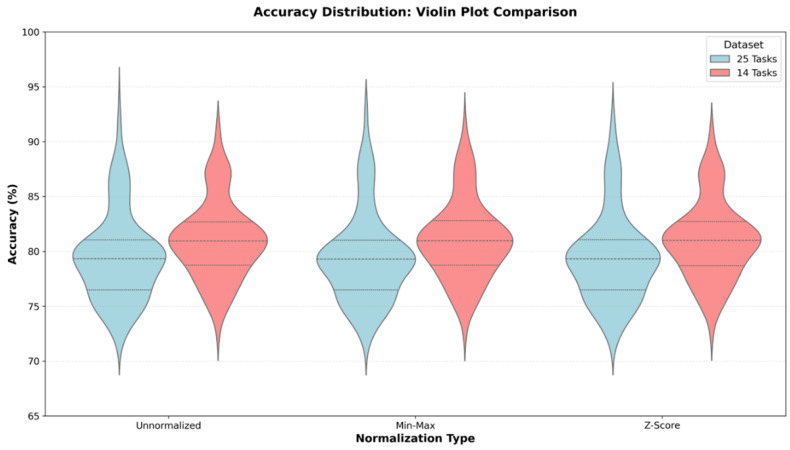
Classification performance distributions for 25-task and 14-task datasets.

To analyze the distribution of classification performance across different model configurations, the results were visualized using violin plots to capture variability, density, and central tendency. The corresponding distributions are shown in Figure 3.

The violin plots show that the reduced 14-task dataset consistently achieved higher accuracy than the full 25-task configuration across all normalization methods. For unnormalized features, the accuracy was 81.03 ± 3.60% for the 14-task dataset and 79.52 ± 4.07% for the 25-task dataset. With Min–Max normalization, the results were 81.05 ± 3.65% and 79.45 ± 4.09%, respectively. Z-Score normalization produced 81.02 ± 3.59% for 14-tasks and 79.45 ± 4.07% for 25-tasks. Approximately 1.5-percentage-point improvement observed after task reduction indicates that eliminating less informative tasks can enhance discriminative capacity. This suggests that a carefully selected subset of tasks may provide a more compact and informative representation than the complete 25-task protocol. In terms of preprocessing, Min–Max normalization resulted in slightly narrower distributions, particularly for the 14-task dataset (SD = 3.65%), indicating marginally improved stability. However, the differences among normalization strategies were negligible (maximum variation < 0.1%), suggesting that feature scaling has limited influence when ensemble classifiers operate on feature sets with identical dimensionality.

The impact of task reduction on computational cost was also evaluated. As shown in Table 28, decreasing the number of features from 1200 to 672 reduced computational load by approximately 44%, training time by 40%, and memory usage by 35%. Statistical analysis also indicated improved average accuracy, F1-score, and standard deviation for the 14-task dataset.

Overall, the results suggest that task reduction improves computational efficiency while maintaining competitive classification performance. The 14-task dataset shows more stable average performance, whereas the 25-task dataset achieves higher peak accuracy under certain configurations.

Table 29 compares the best-performing model configurations obtained using different feature sets.

The results indicate that increasing the number of features from 18 to 48 substantially improves classification performance for the 25-task dataset, with the highest accuracy (94.20%) achieved by the Hard Ensemble classifier combined with L1 Regularization using 110 features. In contrast, the 18-feature configuration yielded a lower best accuracy of 89.87%, obtained with the Hard Ensemble and RF Importance method under both unnormalized and Min–Max normalization methods. When the number of tasks was reduced to 14, the best accuracy decreased slightly to 92.16%, achieved using Min–Max normalization and the Hard Ensemble with L1 Regularization. Overall, these results suggest that richer feature representations contribute to higher peak performance, while task reduction leads to a moderate decrease in maximum accuracy despite improved efficiency.

### 4.6. Comparison with Previous Studies Using the DARWIN Dataset

A comparative overview of prior results and those obtained in this study across different feature and task configurations is presented in Table 30.

In [7], which introduced the DARWIN dataset in 2022, an accuracy of 88.29% was achieved using a Random Forest classifier on an 18-feature, 25-task configuration. Later studies, such as [8,41], published in 2025 and 2024, used single classifiers like CatBoost and Decision Tree, reporting accuracies of 80.81% and 89.0%. Other recent research from the same period has reported even higher classification accuracies: Saha et al. [57] achieved 99.3% using a complex stacking ensemble; Mitra and Rehman [58] and Öcal [59] both reported 97.14% using boosted ensemble models; and Demircioğlu [56] reached 96.23% with a SHAP-integrated SVM approach.

While these studies primarily focus on accuracy, they are based on the original DARWIN dataset structure centered around the standard 18-feature and 25-task configuration without introducing additional feature sets or applying task reduction strategies. In contrast, this study incorporates 30 additional features alongside the baseline set, resulting in an enriched feature space. Of these, 26 had been previously reported in handwriting analysis research but had not been used in DARWIN-based AD diagnosis studies. Notably, the features used in [8,41] partially overlap with the baseline 18 features and include 4 demographic attributes. However, our study’s extended feature set not only encompasses all such prior features but also introduces new ones not previously applied to the DARWIN dataset.

Regarding task configurations, study [8] expanded the task set to 34 by splitting certain multi-word items into individual components, effectively reusing content from the original 25-task set. Similarly, study [41] focused on six tasks selected from the existing set based on semantic and phonological distinctions. These approaches do not reduce the task set itself but instead segment or filter within the original structure. The proposed framework includes a task reduction strategy, demonstrating that high performance can be achieved with only 14 handwriting tasks while reducing memory usage and training time. The highest accuracy of 94.20% was achieved using the 25-task set with enriched features and a hard ensemble strategy.

Although numerically lower than some previously reported values, the present study provides a more balanced and practical solution. It combines robust performance with feature interpretability, computational efficiency, and clinical relevance highlighting its broader utility beyond accuracy alone.

## 5. Conclusions

This study extended the feature representation of the DARWIN dataset by introducing additional handwriting descriptors and systematically evaluating their contribution to Alzheimer’s disease (AD) classification. Experimental analyses were conducted using different task configurations, feature selection strategies, normalization methods, and classification models to examine their impact on performance and computational efficiency. The results indicate that reducing the number of handwriting tasks can preserve competitive classification performance while improving computational efficiency, highlighting the importance of task optimization in handwriting-based diagnostic frameworks.

Feature selection analysis identified several highly informative descriptors, particularly features capturing non-linear movement behavior, entropy-based complexity, and dynamic signal characteristics. Among these, SNRCE_X_ exhibited consistently high selection frequency and strong discriminative capacity. To the best of our knowledge, this study represents the first investigation of SNRCE_X_ in the context of AD diagnosis, suggesting that signal-level measures reflecting structured movement relative to noise may provide valuable diagnostic information. The newly introduced features demonstrated high selection rates across experimental settings, indicating their relevance for capturing subtle motor and cognitive alterations reflected in handwriting behavior.

The task reduction experiments showed that decreasing the number of handwriting tasks reduces feature dimensionality, training time, and memory usage without substantial loss in average classification performance. These findings suggest that carefully designed task subsets may achieve a favorable balance between diagnostic accuracy and computational cost. From a clinical perspective, reducing the assessment from 25 to 14 tasks may shorten evaluation duration and decrease patient burden while maintaining analytical reliability. In addition, the feature-based modeling framework supports interpretability by allowing direct examination of handwriting characteristics contributing to classification outcomes.

Methodologically, the findings emphasize that increasing the number of tasks does not necessarily lead to improved performance and highlight the importance of targeted task design combined with appropriate feature selection and normalization strategies. The modular structure of the framework also enables integration of additional feature representations or classification models in future research.

Several directions remain for further investigation. Future studies will evaluate advanced normalization techniques, including Robust Scaler, MaxAbs Scaler, and power transformations such as Box–Cox and Yeo–Johnson, to examine their effects on robustness and generalization. Additional dimensionality reduction approaches, including PCA, t-SNE, UMAP, and autoencoder-based methods, may also be explored while maintaining interpretability considerations. Moreover, systematic evaluation of alternative task combinations and identification of minimal task sets may further improve efficiency and clinical applicability of handwriting-based AD diagnosis.

Taken together, the proposed framework advances handwriting-based AD analysis by integrating interpretable feature design with efficient task selection, providing a scalable foundation for future clinically oriented digital assessment systems.

## Figures and Tables

**Table 1:** The 48 handwriting features used for the diagnosis of AD.

No	Feature Name	Subject of the Relevant Study	No	Feature Name	Subject of the Relevant Study
1	Total Time	AD [7]	25	Mean Stroke Width	PD [29]
2	Air Time	AD [7]	26	Mean Centroid Distance	CD [30,31]
3	Paper Time	AD [7]	27	Mean Min. Dist. between Strokes	CD [30,31]
4	Mean Speed on paper	AD [7]	28	Mean Stroke Endpoint Distance	CD [30,31]
5	Mean Speed in air	AD [7]	29	Mean Vertical Alignment	CD [30,31]
6	Mean Acceleration on paper	AD [7]	30	Mean Horizontal Alignment	CD [30,31]
7	Mean Acceleration in air	AD [7]	31	Horizontal Shannon Entropy	PD [29,32,33]
8	Mean Jerk on paper	AD [7]	32	Vertical Shannon Entropy	PD [29,32,33]
9	Mean Jerk in air	AD [7]	33	Horizontal Rényi Entropy (2)	PD [29,32,33]
10	Pressure Mean	AD [7]	34	Horizontal Rényi Entropy (3)	PD [29,32,33]
11	Pressure Variation	AD [7]	35	Vertical Rényi Entropy (2)	PD [29,32,33]
12	GMRT on paper	AD [7]	36	Vertical Rényi Entropy (3)	PD [29,32,33]
13	GMRT in air	AD [7]	37	*X*-axis Total Energy	PD [32,33]
14	Mean GMRT	AD [7]	38	*Y*-axis Total Energy	PD [32,33]
15	Pendowns Number	AD [7]	39	*X*-axis Teager–Kaiser Energy	PD [32,33]
16	Max X Extension	AD [7]	40	*Y*-axis Teager–Kaiser Energy	PD [32,33]
17	Max Y Extension	AD [7]	41	*X*-axis CE Signal-to-Noise Ratio	PD [32,33]
18	Dispersion Index	AD [7]	42	*Y*-axis CE Signal-to-Noise Ratio	PD [32,33]
19	Mean Azimuth	CD [34], MCI [35], ESR [36], SR [37]	43	*X*-axis TKE Signal-to-Noise Ratio	PD [32,33]
20	Mean Slope	SR [38]	44	*Y*-axis TKE Signal-to-Noise Ratio	PD [32,33]
21	Total Displacement	PD [29]	45	Speed Std Dev	PD [39]
22	Horizontal Displacement	PD [29]	46	Pressure Std Dev	HDA [40]
23	Vertical Displacement	PD [29]	47	Altitude	MCI [35], ESR [36], SR [37], PD [29]
24	Mean Stroke Height	PD [29]	48	Horizontal Intrinsic Shannon Entropy	PD [29]

AD: Alzheimer’s disease, PD: Parkinson’s disease, SR: Signature recognition, CD: Clock drawing, MCI: Mild cognitive impairment, ESR: Emotional state recognition, HDA: Handwritten document analysis.

**Table 2:** Comparison of Features in Datasets.

ID	Feature Name	[7]	[41]	[8]	This Study	ID	Feature Name	[7]	[41]	[8]	This Study
1	TT	+	-	+	+	25	MSW	-	-	-	+
2	AT	+	-	-	+	26	MCD	-	-	-	+
3	PT	+	-	-	+	27	MMDS	-	-	-	+
4	MSP	+	-	-	+	28	MSED	-	-	-	+
5	MSA	+	-	-	+	29	MVA	-	-	-	+
6	MAP	+	-	-	+	30	MHA	-	-	-	+
7	MAA	+	-	-	+	31	HSE	-	-	-	+
8	MJP	+	-	-	+	32	VSE	-	-	-	+
9	MJA	+	-	-	+	33	HRE_2_	-	-	-	+
10	PM	+	+	+	+	34	HRE_3_	-	-	-	+
11	PV	+	-	-	+	35	VRE_2_	-	-	-	+
12	GMRTP	+	-	-	+	36	VRE_3_	-	-	-	+
13	GMRTA	+	-	-	+	37	CE_X_	-	-	-	+
14	GMRT	+	-	-	+	38	CE_Y_	-	-	-	+
15	PWN	+	+	+	+	39	TKE_X_	-	-	-	+
16	XE	+	+	+	+	40	TKE_Y_	-	-	-	+
17	YE	+	+	+	+	41	SNRCE_X_	-	-	-	+
18	DI	+	-	-	+	42	SNRCE_Y_	-	-	-	+
19	MA	-	+	+	+	43	SNRTKE_X_	-	-	-	+
20	MS	-	+	+	+	44	SNRTKE_Y_	-	-	-	+
21	TD	-	+	+	+	45	SSD	-	-	-	+
22	HD	-	-	-	+	46	PSD	-	-	-	+
23	VD	-	-	-	+	47	Altitude	-	-	-	+
24	MSH	-	-	-	+	48	H_IMF1_	-	-	-	+

TT: Total time, AT: Air time, PT: Paper time, MSP: Mean Speed on paper, MSA: Mean Speed in air. MAP: Mean Acceleration on paper, MAA: Mean Acceleration in air, MJP: Mean Jerk on paper, MJA: Mean Jerk in air, PM: Pressure Mean, PV: Pressure Variation, GMRTP: GMRT on paper, GMRTA: GMRT in air, GMRT: Mean GMRT, PWN: Pendowns Number, XE: Max X Extension, YE: Max Y Extension, DI: Dispersion Index.

**Table 3:** Task-wise Distribution of Empty Files.

Task Number	Total Empty Files	Healthy Empty	Patient Empty	Task Number	Total Empty Files	Healthy Empty	Patient Empty
19	28	6	22	16	4	2	2
21	13	3	10	17	4	2	2
25	12	2	10	18	4	2	2
20	10	2	8	8	4	4	0
22	10	2	8	2	3	3	0
24	10	2	8	6	3	2	1
23	9	2	7	9	3	2	1
13	6	2	4	3	2	1	1
14	6	2	4	7	2	2	0
12	5	3	2	4	1	1	0
15	5	2	3	5	1	1	0
10	4	2	2	1	0	0	0
11	4	2	2				

**Table 4:** Selection Frequencies of Tasks.

Task No	Unnormalized	Z-Score	Min–Max	Task No	Unnormalized	Z-Score	Min–Max
9	97	109	97	20	13	13	13
7	33	23	33	3	13	13	13
8	31	30	31	5	13	13	13
10	20	19	20	24	12	13	12
13	19	19	19	1	11	11	11
14	19	19	19	21	11	11	11
23	17	16	17	25	11	11	11
4	16	16	16	18	10	10	10
12	16	16	16	17	9	9	9
16	16	16	16	19	7	7	7
11	15	15	15	22	4	4	4
2	14	14	14	15	0	0	0
6	13	13	13				

**Table 5:** Average Selection Frequencies of Features Across Different Normalization Strategies (25-task).

Feature	Unnormalized	Z-Score	Min–Max	Overall Average	Feature	Unnormalized	Z-Score	Min–Max	Overall Average
**SNRCE_X_**	6.75	6.75	6.80	6.77	GMRTP	2.00	2.00	2.40	2.13
**MA**	5.25	5.25	5.40	5.30	**VSE**	2.00	2.25	2.00	2.08
**MS**	4.50	4.50	4.60	4.53	**MCD**	2.00	1.75	2.40	2.05
**MVA**	4.25	4.50	4.60	4.45	**H_IMF1_**	1.75	1.75	2.00	1.83
**Altitude**	4.25	4.00	4.60	4.28	PT	1.75	2.00	1.60	1.78
MJA	4.00	4.00	4.20	4.07	**MSED**	1.75	1.75	1.80	1.77
**TKE_X_**	4.00	3.75	4.00	3.92	**HRE_2_**	1.50	1.75	2.00	1.75
YE	3.50	3.50	4.00	3.67	**VRE_3_**	1.50	1.75	1.80	1.68
**MSW**	3.50	3.50	3.80	3.60	**SNRCE_Y_**	1.50	1.50	2.00	1.67
GMRTA	3.50	3.25	4.00	3.58	**VD**	1.50	1.25	1.60	1.45
**HSE**	3.25	3.50	3.40	3.38	**CE_Y_**	1.50	1.25	1.60	1.45
**SNRTKE_Y_**	3.25	3.00	3.20	3.15	PWN	1.25	1.00	2.00	1.42
MJP	3.00	3.00	3.20	3.07	MAA	1.25	1.50	1.20	1.32
**PSD**	3.00	3.00	3.00	3.00	DI	1.00	1.00	1.40	1.13
**MMDS**	2.75	2.75	3.20	2.90	**VRE_2_**	1.00	1.25	1.00	1.08
PV	3.00	2.50	3.20	2.90	MSP	1.00	1.25	1.00	1.08
**MHA**	2.75	2.75	2.80	2.77	**CE_X_**	1.00	1.00	1.20	1.07
**MSH**	2.50	2.50	3.00	2.67	**TKE_Y_**	1.00	1.00	1.20	1.07
AT	2.50	2.75	2.60	2.62	MAP	0.75	1.00	0.80	0.85
MSA	2.25	2.50	2.20	2.32	TT	0.75	0.75	0.80	0.77
XE	2.25	2.00	2.40	2.22	**HD**	0.75	0.75	0.80	0.77
**HRE_3_**	2.00	2.25	2.20	2.15	**SSD**	0.75	0.50	0.60	0.62
GMRT	2.00	2.00	2.40	2.13	PM	0.50	0.25	0.80	0.52
**SNRTKE_X_**	2.00	2.00	2.40	2.13	**TD**	0.25	0.25	0.80	0.43

Features shown in bold represent those investigated for AD diagnosis in this study.

**Table 6:** Average Selection Frequencies of Features Across Different Normalization Strategies (14-task).

Feature	Unnormalized	Z-Score	Min–Max	Overall Average	Feature	Unnormalized	Z-Score	Min–Max	Overall Average
**SNRCE_X_**	6.75	6.75	6.75	6.75	GMRTP	2.00	2.00	2.00	2.00
**MA**	5.25	5.25	5.25	5.25	**SNRTKE_X_**	2.00	2.00	2.00	2.00
**MS**	4.50	4.50	4.50	4.50	**MCD**	2.00	1.75	2.00	1.92
**MVA**	4.25	4.50	4.25	4.33	PT	1.75	2.00	1.75	1.83
**Altitude**	4.25	4.00	4.25	4.17	**MSED**	1.75	1.75	1.75	1.75
MJA	4.00	4.00	4.00	4.00	**H_IMF1_**	1.75	1.75	1.75	1.75
**TKE_X_**	4.00	3.75	4.00	3.92	**VRE_3_**	1.50	1.75	1.50	1.58
**MSW**	3.50	3.50	3.50	3.50	**HRE_2_**	1.50	1.75	1.50	1.58
YE	3.50	3.50	3.50	3.50	**SNRCE_Y_**	1.50	1.50	1.50	1.50
GMRTA	3.50	3.25	3.50	3.42	**VD**	1.50	1.25	1.50	1.42
**HSE**	3.25	3.50	3.25	3.33	**CE_Y_**	1.50	1.25	1.50	1.42
**SNRTKE_Y_**	3.25	3.00	3.25	3.17	MAA	1.25	1.50	1.25	1.33
**PSD**	3.00	3.00	3.00	3.00	PWN	1.25	1.00	1.25	1.17
MJP	3.00	3.00	3.00	3.00	MSP	1.00	1.25	1.00	1.08
PV	3.00	2.50	3.00	2.83	**VRE_2_**	1.00	1.25	1.00	1.08
**MHA**	2.75	2.75	2.75	2.75	DI	1.00	1.00	1.00	1.00
**MMDS**	2.75	2.75	2.75	2.75	**TKE_Y_**	1.00	1.00	1.00	1.00
AT	2.50	2.75	2.50	2.58	**CE_X_**	1.00	1.00	1.00	1.00
**MSH**	2.50	2.50	2.50	2.50	MAP	0.75	1.00	0.75	0.83
MSA	2.25	2.50	2.25	2.33	**HD**	0.75	0.75	0.75	0.75
XE	2.25	2.00	2.25	2.17	TT	0.75	0.75	0.75	0.75
**HRE_3_**	2.00	2.25	2.00	2.08	**SSD**	0.75	0.50	0.75	0.67
**VSE**	2.00	2.25	2.00	2.08	PM	0.50	0.25	0.50	0.42
GMRT	2.00	2.00	2.00	2.00	**TD**	0.25	0.25	0.25	0.25

Features shown in bold represent those investigated for AD diagnosis in this study.

**Table 7:** Performance of Top 15 Model Configurations on Unnormalized Dataset (25 Tasks).

Classifier	Feature Selection	*k*	Accuracy (%)	F1 Score (%)	Sensitivity (%)	Specificity (%)	Std Accuracy
Hard Ensemble	L1 Regularization	110	94.20	94.19	94.10	94.44	4.81
Hard Ensemble	L1 Regularization	120	91.96	91.91	97.78	86.67	3.11
Hard Ensemble	L1 Regularization	125	91.89	91.84	95.00	88.89	6.63
Hard Ensemble	L1 Regularization	145	91.42	91.41	94.10	88.89	7.22
Hard Ensemble	L1 Regularization	115	90.65	90.65	90.28	91.11	6.75
Hard Ensemble	RF Importance	150	89.33	89.26	91.90	87.04	4.57
Hard Ensemble	L1 Regularization	105	88.56	88.44	90.97	86.11	4.55
Hard Ensemble	L1 Regularization	100	88.50	88.31	97.78	80.00	5.73
Hard Ensemble	RF Importance	130	88.43	88.35	90.56	86.67	4.28
Hard Ensemble	RFE Random Forest	110	88.43	88.41	90.56	86.67	4.28
Hard Ensemble	L1 Regularization	140	88.43	88.30	87.78	88.89	7.28
Hard Ensemble	L1 Regularization	130	88.40	88.32	93.75	83.33	8.99
Hard Ensemble	RFE Random Forest	145	88.32	88.27	94.10	83.33	4.94
Hard Ensemble	L1 Regularization	135	87.47	87.34	89.81	85.19	5.71
Hard Ensemble	RFE Random Forest	115	87.32	87.29	88.33	86.67	4.82

**Table 8:** Average Performance of Classifiers on Unnormalized Dataset (25 Tasks).

Classifier	Mean Acc (%)	Std Acc	Max Acc (%)	Min Acc (%)	Mean F1 (%)	Mean Sens (%)	Mean Spec (%)
Hard Ensemble	87.13	2.27	94.20	83.79	87.07	90.36	84.33
XGBoost	80.77	1.92	84.54	76.96	80.57	81.26	80.15
RandomForest	80.38	1.17	83.40	78.20	80.18	81.51	79.18
Soft Ensemble	79.00	1.33	81.11	76.47	78.67	82.77	75.35
	76.84	1.92	81.01	72.97	76.42	81.65	72.19
SVM	76.70	2.49	80.42	72.39	76.29	80.80	72.79
MLPClassifier	75.79	2.18	81.08	71.34	75.24	81.61	70.19

**Table 9:** Average Performance of Feature Selection Methods on Unnormalized Dataset (25 Tasks).

Feature Selection	Mean Acc (%)	Std Acc	Max Acc (%)	Min Acc (%)	Mean F1 (%)	Mean Sens (%)	Mean Spec (%)
L1 Regularization	80.90	4.24	94.20	73.69	80.62	84.22	77.66
RF Importance	80.28	3.72	89.32	71.34	80.00	83.01	77.70
RFE RandomForest	78.52	4.12	88.43	72.39	78.17	82.20	75.03
XGB Importance	78.36	3.62	86.85	73.04	78.03	81.97	74.86

**Table 10:** Performance of Top 15 Model Configurations on Min–Max-Normalized Dataset (25-Task).

Classifier	Feature Selection	*k*	Accuracy (%)	F1 Score (%)	Sensitivity (%)	Specificity (%)	Std Accuracy
Hard Ensemble	L1 Regularization	120	93.07	93.05	97.78	88.89	2.71
Hard Ensemble	L1 Regularization	100	92.89	92.77	100.00	86.11	6.95
Hard Ensemble	L1 Regularization	110	92.81	92.80	94.10	91.67	3.05
Hard Ensemble	L1 Regularization	135	90.72	90.65	95.00	86.67	6.66
Hard Ensemble	RFE Random Forest	150	90.31	90.30	92.13	88.89	6.89
Hard Ensemble	L1 Regularization	150	89.71	89.67	87.50	91.67	7.40
Hard Ensemble	L1 Regularization	125	89.67	89.55	92.78	86.67	6.25
Hard Ensemble	RF Importance	150	89.54	89.46	92.78	86.67	5.08
Hard Ensemble	L1 Regularization	105	88.56	88.44	90.97	86.11	4.55
Hard Ensemble	L1 Regularization	115	88.43	88.40	90.28	86.67	4.28
Hard Ensemble	RF Importance	115	88.43	88.35	93.06	84.44	5.97
Hard Ensemble	RFE Random Forest	110	88.43	88.41	90.56	86.67	4.28
Hard Ensemble	RF Importance	130	88.43	88.35	90.56	86.67	4.28
Hard Ensemble	L1 Regularization	145	88.40	88.29	91.90	85.19	5.34
Hard Ensemble	XGB Importance	145	88.34	88.31	92.13	85.19	6.05

**Table 11:** Average Performance of Classifiers on Min–Max-Normalized Dataset (25 Tasks).

Classifier	Mean Acc (%)	Std Acc	Max Acc (%)	Min Acc (%)	Mean F1 (%)	Mean Sens (%)	Mean Spec (%)
Hard Ensemble	87.25	2.48	93.07	82.74	87.19	90.44	84.48
XGBoost	80.61	1.62	83.99	77.61	80.44	81.58	79.53
RandomForest	80.18	1.11	83.40	77.65	79.99	81.68	78.65
Soft Ensemble	78.82	1.34	81.63	75.92	78.51	82.38	75.38
-	76.86	1.94	81.01	72.97	76.43	81.70	72.16
SVM	76.67	2.46	80.42	72.39	76.26	80.78	72.77
MLPClassifier	75.74	2.07	81.08	71.34	75.18	81.53	70.16

**Table 12:** Average Performance of Feature Selection Methods on Min–Max-Normalized Dataset (25 Tasks).

Feature Selection	Mean Acc (%)	Std Acc	Max Acc (%)	Min Acc (%)	Mean F1 (%)	Mean Sens (%)	Mean Spec (%)
L1 Regularization	80.90	4.22	93.07	73.69	80.61	84.47	77.40
RF Importance	80.00	3.60	89.54	71.34	79.72	82.90	77.26
RFE RandomForest	78.61	4.36	90.30	72.39	78.28	82.38	75.05
XGB Importance	78.27	3.62	88.34	73.04	77.96	81.73	74.93

**Table 13:** Performance of Top 15 Model Configurations on Z-score-Normalized Dataset (25-Task).

Classifier	Feature Selection	*k*	Accuracy (%)	F1 Score (%)	Sensitivity (%)	Specificity (%)	Std Accuracy
Hard Ensemble	L1 Regularization	110	92.81	92.80	94.10	91.67	3.05
Hard Ensemble	L1 Regularization	120	91.96	91.91	97.78	86.67	3.11
Hard Ensemble	L1 Regularization	100	91.42	91.28	100.00	83.33	7.22
Hard Ensemble	L1 Regularization	135	90.72	90.65	95.00	86.67	6.66
Hard Ensemble	L1 Regularization	115	90.65	90.65	90.28	91.11	6.75
Hard Ensemble	RFE Random Forest	150	90.31	90.30	92.13	88.89	6.89
Hard Ensemble	L1 Regularization	150	89.71	89.67	87.50	91.67	7.40
Hard Ensemble	RF Importance	150	89.54	89.46	92.78	86.67	5.08
Hard Ensemble	L1 Regularization	105	88.56	88.44	90.97	86.11	4.55
Hard Ensemble	L1 Regularization	125	88.50	88.37	90.28	86.67	5.73
Hard Ensemble	RFE Random Forest	110	88.43	88.41	90.56	86.67	4.28
Hard Ensemble	RF Importance	130	88.43	88.35	90.56	86.67	4.28
Hard Ensemble	RF Importance	115	88.43	88.35	93.06	84.44	5.97
Hard Ensemble	L1 Regularization	130	88.40	88.37	91.90	85.19	7.50
Hard Ensemble	RFE Random Forest	125	88.32	88.27	94.10	83.33	4.94

**Table 14:** Average Performance of Classifiers on Z-Score-Normalized Dataset (25 Tasks)

Classifier	Mean Acc (%)	Std Acc	Max Acc (%)	Min Acc (%)	Mean F1 (%)	Mean Sens (%)	Mean Spec (%)
Hard Ensemble	87.14	2.41	92.81	82.48	87.08	90.27	84.43
XGBoost	80.61	1.61	83.99	77.61	80.45	81.58	79.53
Random Forest	80.36	1.25	83.40	77.61	80.17	81.54	79.12
Soft Ensemble	78.81	1.34	81.11	76.44	78.50	82.38	75.35
LogisticRegression	76.73	1.95	81.01	72.29	76.30	81.52	72.09
SVM	76.71	2.51	80.42	72.39	76.30	80.83	72.79
MLPClassifier	75.78	2.11	81.08	71.34	75.22	81.61	70.16

**Table 15:** Average Performance of Feature Selection Methods on Z-score-Normalized Dataset (25 Tasks).

Feature Selection	Mean Acc (%)	Std Acc	Max Acc (%)	Min Acc (%)	Mean F1 (%)	Mean Sens (%)	Mean Spec (%)
L1 Regularization	80.90	4.15	92.81	73.69	80.63	84.35	77.53
RF Importance	80.09	3.65	89.54	71.34	79.80	82.95	77.37
RFE Random Forest	78.60	4.36	90.30	72.29	78.26	82.41	74.99
XGB Importance	78.20	3.55	86.85	73.04	77.89	81.56	74.94

**Table 16:** Performance Comparison of Optimal Model Configurations Across Normalization Strategies (25 Tasks).

Normalization	Best Acc (%)	Best F1	*k* Features	Sensitivity (%)	Specificity (%)	Sens-Spec Balance	Most Stable Std	Mean Acc
Unnormalized	94.20	94.19	110	94.10	94.44	0.35	3.11	79.52
Min–Max	93.07	93.05	120	97.78	88.89	8.89	2.71	79.45
Z-Score	92.81	92.80	110	94.10	91.67	2.43	3.05	79.45

**Table 17:** Performance of Top 15 Model Configurations on Unnormalized Dataset (14 Tasks).

Classifier	Feature Selection	*k*	Accuracy (%)	F1 Score (%)	Sensitivity (%)	Specificity (%)	Std Accuracy
Hard Ensemble	RFE Random Forest	150	91.42	91.38	88.54	94.44	3.31
Hard Ensemble	RF Importance	125	91.26	91.23	96.88	86.11	3.49
Hard Ensemble	L1 Regularization	145	91.23	91.10	87.73	94.44	6.20
Hard Ensemble	RF Importance	130	90.36	90.32	96.06	85.19	5.83
Hard Ensemble	RFE Random Forest	125	89.48	89.46	92.78	86.67	5.00
Hard Ensemble	RFE Random Forest	120	89.38	89.36	92.13	87.04	4.48
Hard Ensemble	L1 Regularization	140	89.26	89.16	89.68	88.89	7.13
Hard Ensemble	L1 Regularization	100	88.45	88.42	90.05	87.04	6.34
Hard Ensemble	RF Importance	150	88.40	88.38	91.90	85.19	6.28
Hard Ensemble	RFE Random Forest	100	88.40	88.40	90.05	87.04	5.34
Hard Ensemble	L1 Regularization	115	88.38	88.22	85.91	90.48	4.88
Hard Ensemble	L1 Regularization	135	88.34	88.23	85.65	90.74	7.54
Hard Ensemble	RF Importance	140	87.81	87.76	90.28	85.71	7.36
Hard Ensemble	L1 Regularization	145	88.40	88.29	91.90	85.19	5.34
Hard Ensemble	XGB Importance	145	88.34	88.31	92.13	85.19	6.05

**Table 18:** Average Performance of Classifiers on Unnormalized Dataset (14 Tasks).

Classifier	Mean Acc (%)	Std Acc	Max Acc (%)	Min Acc (%)	Mean F1 (%)	Mean Sens (%)	Mean Spec (%)
Hard Ensemble	87.55	1.54	91.42	84.31	87.48	89.22	96.88
XGBoost	82.05	1.41	85.13	79.38	81.77	82.78	87.08
Soft Ensemble	81.50	1.74	84.51	78.20	81.21	84.02	88.47
Random Forest	81.27	1.12	84.02	79.35	81.10	81.02	84.86
SVM	79.62	1.71	82.71	76.44	79.33	82.49	88.33
MLPClassifier	76.62	2.03	82.16	72.35	76.05	82.02	89.31
LogisticRegression	78.59	2.01	81.60	74.84	78.28	82.75	90.56

**Table 19:** Average Performance of Feature Selection Methods on Unnormalized Dataset (14 Tasks).

Feature Selection	Mean Acc (%)	Std Acc	Max Acc (%)	Min Acc (%)	Mean F1 (%)	Mean Sens (%)	Mean Spec (%)
RFE RandomForest	80.40	3.75	91.42	72.35	80.17	82.43	78.46
RF Importance	81.49	3.60	91.26	74.77	81.15	85.77	77.36
L1 Regularization	82.27	3.08	91.23	75.36	82.03	83.32	81.17
XGB Importance	79.95	3.54	87.49	72.45	79.64	82.36	77.49

**Table 20:** Performance of Top 15 Model Configurations on Min–Max-Normalized Dataset (14 Tasks).

Classifier	Feature Selection	*k*	Accuracy (%)	F1 Score (%)	Sensitivity (%)	Specificity (%)	Std Accuracy
Hard Ensemble	L1 Regularization	120	92.16	92.03	91.67	92.59	8.04
Hard Ensemble	RFE Random Forest	150	91.42	91.38	88.54	94.44	3.31
Hard Ensemble	RF Importance	130	90.36	90.32	96.06	85.19	5.83
Hard Ensemble	RFE Random Forest	120	90.31	90.29	93.98	87.04	6.04
Hard Ensemble	RF Importance	125	89.79	89.76	93.75	86.11	3.10
Hard Ensemble	RF Importance	120	89.41	89.35	92.50	86.67	6.44
Hard Ensemble	L1 Regularization	125	89.38	89.36	90.05	88.89	5.82
Hard Ensemble	L1 Regularization	140	89.26	89.16	89.68	88.89	7.13
Hard Ensemble	L1 Regularization	145	89.08	88.94	89.29	88.89	9.26
Hard Ensemble	L1 Regularization	105	88.45	88.42	90.05	87.04	6.34
Hard Ensemble	XGB Importance	145	88.43	88.38	90.56	86.67	4.28
Hard Ensemble	XGB Importance	140	88.43	88.42	88.06	88.89	7.01
Hard Ensemble	RF Importance	150	88.40	88.38	91.90	85.19	6.28
Hard Ensemble	L1 Regularization	115	88.38	88.22	85.91	90.48	4.88
Hard Ensemble	L1 Regularization	110	88.38	88.31	85.91	90.48	5.73

**Table 21:** Average Performance of Classifiers on Min–Max-Normalized Dataset (14 Tasks).

Classifier	Mean Acc (%)	Std Acc	Max Acc (%)	Min Acc (%)	Mean F1 (%)	Mean Sens (%)	Mean Spec (%)
Hard Ensemble	87.63	1.69	92.16	84.71	87.57	88.96	86.47
XGBoost	81.96	1.67	85.16	78.72	81.68	83.15	80.71
Soft Ensemble	81.44	1.72	84.51	78.20	81.14	83.93	78.91
RandomForest	81.52	1.16	83.43	79.35	81.36	81.53	81.50
SVM	79.62	1.71	82.71	76.44	79.33	82.49	76.72
MLPClassifier	76.62	2.03	82.16	72.35	76.05	82.02	71.37
LogisticRegression	78.59	2.01	81.60	74.84	78.28	82.75	74.46

**Table 22:** Average Performance of Feature Selection Methods on Min–Max-Normalized Dataset (14 Tasks).

Feature Selection	Mean Acc (%)	Std Acc	Max Acc (%)	Min Acc (%)	Mean F1 (%)	Mean Sens (%)	Mean Spec (%)
L1 Regularization	82.49	3.18	92.16	75.36	82.25	83.81	81.11
RFE RandomForest	80.43	3.78	91.42	72.35	80.20	82.30	78.62
RF Importance	81.37	3.56	90.36	74.77	81.04	85.62	77.26
XGB Importance	79.93	3.58	88.43	72.45	79.62	82.45	77.38

**Table 23:** Performance of Top 15 Model Configurations on Z-score-Normalized Dataset (14 task).

Classifier	Feature Selection	*k*	Accuracy (%)	F1 Score (%)	Sensitivity (%)	Specificity (%)	Std Accuracy
Hard Ensemble	RF Importance	125	91.26	91.23	96.88	86.11	3.49
Hard Ensemble	L1 Regularization	145	91.23	91.10	87.73	94.44	6.20
Hard Ensemble	RF Importance	130	90.36	90.32	96.06	85.19	5.83
Hard Ensemble	RFE Random Forest	150	89.95	89.91	88.54	91.67	5.67
Hard Ensemble	RFE Random Forest	125	89.48	89.46	92.78	86.67	5.00
Hard Ensemble	L1 Regularization	110	89.22	89.17	87.70	90.48	5.09
Hard Ensemble	L1 Regularization	140	88.47	88.32	88.09	88.89	7.48
Hard Ensemble	L1 Regularization	115	88.44	88.29	84.90	91.67	4.52
Hard Ensemble	L1 Regularization	125	88.40	88.35	85.88	90.74	3.83
Hard Ensemble	RF Importance	150	88.40	88.38	91.90	85.19	6.28
Hard Ensemble	RFE Random Forest	120	88.40	88.40	90.05	87.04	5.34
Hard Ensemble	L1 Regularization	135	88.34	88.23	85.65	90.74	7.54
Hard Ensemble	XGB Importance	100	88.23	88.04	84.38	91.67	8.32
Hard Ensemble	RF Importance	140	87.81	87.76	90.28	85.71	7.36
Hard Ensemble	RF Importance	135	87.72	87.58	88.29	87.30	7.52

**Table 24:** Average Performance of Classifiers on Z-score-Normalized Dataset (14 task).

Classifier	Mean Acc (%)	Std Acc	Max Acc (%)	Min Acc (%)	Mean F1 (%)	Mean Sens (%)	Mean Spec (%)
Hard Ensemble	87.41	1.55	91.26	84.22	87.33	89.00	86.01
XGBoost	82.08	1.51	86.34	79.38	81.80	82.80	81.26
RandomForest	81.34	1.35	84.58	78.27	81.19	81.14	81.53
Soft Ensemble	81.47	1.77	84.54	77.61	81.18	84.04	78.86
SVM	79.58	1.66	82.71	76.44	79.29	82.46	76.67
MLP Classifier	76.59	2.03	81.60	72.35	76.01	82.17	71.17
LogisticRegression	78.64	2.07	81.60	74.84	78.33	82.78	74.54

**Table 25:** Average Performance of Feature Selection Methods on Z-score-Normalized Dataset (14 task).

Feature Selection	Mean Acc (%)	Std Acc	Max Acc (%)	Min Acc (%)	Mean F1 (%)	Mean Sens (%)	Mean Spec (%)
RF Importance	81.40	3.57	91.26	74.77	81.06	85.78	77.16
L1 Regularization	82.35	3.07	91.23	75.36	82.10	83.44	81.20
RFE RandomForest	80.28	3.65	89.95	72.35	80.05	82.32	78.32
XGB Importance	80.04	3.62	88.24	72.45	79.73	82.40	77.63

**Table 26:** Performance Comparison of Optimal Model Configurations Across Normalization Strategies (14 task).

Normalization	Best Acc (%)	Best F1	*k* Features	Sensitivity (%)	Specificity (%)	Sens-Spec Balance	Most Stable Std	Mean Acc
Unnormalized	91.42	91.38	150	88.54	94.44	5.90	2.90	81.03
Min–Max	92.16	92.03	120	91.67	92.59	0.93	2.90	81.05
Z-Score	91.26	91.23	125	96.88	86.11	10.76	2.90	81.02

**Table 27:** Comparison of Optimal Model Performance on 25-Task and 14-Task Datasets.

Dataset	Normalization	Mean Accuracy (%)	Best Accuracy (%)	Best Model	*k*
25-Task	Unnormalized	79.52 ± 4.07	94.20	Hard Ensemble + L1	110
25-Task	Min–Max	79.45 ± 4.09	93.07	Hard Ensemble + L1	120
25-Task	Z-Score	79.45 ± 4.07	92.81	Hard Ensemble + L1	110
14-Task	Unnormalized	81.03 ± 3.60	91.42	Hard Ensemble + RFE	150
14-Task	Min–Max	81.05 ± 3.65	92.16	Hard Ensemble + L1	120
14-Task	Z-Score	81.02 ± 3.59	91.26	Hard Ensemble + RF	125

**Table 28:** Comparison of the Calculation Cost and Performance Effects of Reducing the Number of Tasks.

Metric	25-Task Dataset	14-Task Dataset	Change (%)
Total Number of Features	1200	672	−44.00%
Average Number of Selected Features	125	125	0
Mean Accuracy	79.47%	81.03%	+1.56%
Maximum Accuracy	94.20%	91.61%	−1.75%
Mean F1-Score	79.16%	80.75%	+1.59%
Standard Deviation	4.08%	3.61%	−11.52%
Training Time (Relative)	100%	~60%	−40%
Memory Usage (Relative)	100%	~65%	−35%

**Table 29:** Performance Comparison of 18- and 48-Feature Sets.

Dataset	Normalization	Best Accuracy (%)	Best Model	*k*
18-Feature–25-Task	Unnormalized, Min–Max	89.87	Hard Ensemble + RF_Importance	125
48-Feature–25-Task	Unnormalized	94.20	Hard Ensemble + L1	110
48-Feature–14-Task	Min–Max	92.16	Hard Ensemble + L1	120

**Table 30:** Comparison of previous studies and the proposed approach on the DARWIN dataset.

Study	Model	# of Features/Task	# of Tasks	Accuracy (%)
Singh & Chaturvedi [25]	Stacking Ensemble	18	25	88.57
Saha et al. [57]	Stacking Ensemble (RF + LR/XGB/LightGBM/CatBoost (CB))	18	25	99.3
Mitra & Rehman [58]	Stacking Ensemble with Feature Selection	18	25	97.14
Öcal [59]	LightGBM + AdaBoost + CatBoost (Hard Voting)	18	25	97.14
Demircioğlu [56]	SHAP + SVM	18	25	96.23
Cilia et al. [7]	RF	18	25	88.29
Nardone et al. [8]	CatBoost	31 + 4 (demographic)	34	80.81
Cilia et al. [41]	Decision Tree (DT)	22 + 4 (demographic)	6	89.00
This study	Hard Ensemble + RF Importance	18	25	89.87
This study	Hard Ensemble + L1	48	25	94.20
This study	Hard Ensemble + L1	48	14	92.16

## Data Availability

The original data presented in the study are openly available in https://doi.org/10.5281/zenodo.18413419.
